# Global Mortality and Disability‐Adjusted Life Years Attributable to Tobacco Exposure Among Middle‐Aged and Older Adults With Type 2 Diabetes, 1990–2021: A Systematic Analysis of GBD 2021 Data With Projections to 2042

**DOI:** 10.1155/jdr/5522115

**Published:** 2026-03-12

**Authors:** Yujun He, Minhui Liu, Bowen Xing, Hui Xu, Jiujie He, Wei Mai, Simin Qin, Zhenyi Luo, Xiaoyi Wang, Yi Xu, Yuping Ye

**Affiliations:** ^1^ Department of Traditional Chinese Medicine, Taizhou Hospital of Zhejiang Province Affiliated to Wenzhou Medical University, Linhai City,Taizhou City, Zhejiang Province, China, wmu.edu.cn; ^2^ Department of Traditional Chinese Medicine, The First People′s Hospital of Chenzhou City, Chenzhou City, Hunan Province, China; ^3^ Faculty of Acupuncture, Moxibustion and Tuina, Guangxi University of Chinese Medicine, Nanning City, Guangxi Province, China, gxtcm.com; ^4^ Department of Traditional Chinese Medicine, Guangxi Medical University Cancer Hospital, Nanning City, Guangxi Province, China, gxmu.edu.cn; ^5^ Rehabilitation Medicine Department, The Second Affiliated Hospital of Hainan Medical University, Haikou City, Hainan Province, China, hainmc.edu.cn; ^6^ College of Traditional Chinese Medicine, Chongqing University of Chinese Medicine, Chongqing City, China

**Keywords:** middle-aged and older adults, tobacco exposure, Type 2 diabetes mellitus

## Abstract

**Background:**

Type 2 diabetes mellitus (T2DM) has emerged as a pressing global health challenge, and tobacco exposure constitutes a major modifiable risk factor—particularly among middle‐aged and elderly populations. However, a comprehensive understanding of the global burden of tobacco exposure–attributable mortality and disability‐adjusted life years (DALYs) in this demographic remains limited.

**Objective:**

This study is aimed at quantifying the global burden of deaths and DALYs attributable to tobacco exposure among middle‐aged and elderly individuals with T2DM from 1990 to 2021. Simultaneously, this study assesses disparities across regions, genders, and age groups; examines associations with the sociodemographic index (SDI); and forecasts trends in disease burden from 2022 to 2042.

**Method:**

Utilizing data from the Global Burden of Disease (GBD) 2021 study, which encompasses 204 countries and territories, we assessed burden counts and rates per 100,000 population among individuals aged 55 years and older. We further computed the estimated annual percentage changes (EAPCs) and applied age–period–cohort (APC) modeling, frontier analysis, and Bayesian age–period–cohort (BAPC) forecasting models to enable comprehensive analysis.

**Results:**

Globally, tobacco exposure–attributable T2DM deaths increased by 101.10%, and DALYs rose by 140.38%, though the mortality rate decreased by 9.13%. Regions with middle and low–middle SDI shouldered the highest burden of tobacco‐attributable T2DM. High‐SDI regions demonstrated the most substantial declines in burden rates, whereas low–middle‐SDI regions experienced the largest relative increases. Males exhibited greater mortality and DALYs counts compared to females, while females showed more pronounced reductions in burden trends. APC modeling further indicated that advancing age correlated with elevated risks of tobacco exposure–attributable T2DM outcomes, whereas younger birth cohorts showed lower risks. Frontier analysis identified middle‐SDI countries (e.g., Kiribati) as exhibiting the greatest deviation from optimal performance. Projections through 2042 indicate that the mortality rate is projected to continue declining: It will reach 9.25 per 100,000 aged ≥ 55 population in 2030 (95% CI: 8.64–9.85) and 8.57 per 100,000 aged ≥ 55 population in 2042 (95% CI: 6.89–10.24).

**Conclusions:**

Tobacco exposure–attributable T2DM burden demonstrates marked variations across geographic regions, sex, and age groups, highlighting the imperative for targeted interventions in low–middle‐SDI regions. Tobacco control strategies from high‐SDI regions serve as scalable models for mitigating this preventable disease burden.

## 1. Introduction

Type 2 diabetes mellitus (T2DM) constitutes 90%–95% of all diabetes mellitus cases and predominates among middle‐aged and older adults [1–3] As a chronic metabolic condition, T2DM significantly compromises patients′ quality of life while incurring substantial economic costs for global healthcare systems. Both mortality and disability‐adjusted life years (DALYs) linked to T2DM have demonstrated persistent upward trends. Moreover, its complications—including cardiovascular diseases, renal failure, and lower‐limb amputations—drive considerable premature mortality and reduced life expectancy [3, 4].

Among the numerous risk factors associated with T2DM pathogenesis, tobacco exposure (encompassing smoking, secondhand smoke, and chewing tobacco) has attracted growing attention due to its complex association with T2DM development. Nicotine, the primary addictive component in tobacco products, has been demonstrated to disrupt insulin signaling pathways, enhance insulin resistance, and perturb lipid metabolism—all of which represent key factors in T2DM progression [5, 6]. Epidemiological studies consistently indicate that individuals exposed to tobacco exhibit a higher incidence of T2DM than those without such exposure [7].

Middle‐aged and older adults represent a particularly vulnerable group in the interplay between T2DM and tobacco exposure. This age demographic demonstrates a higher T2DM prevalence than younger cohorts and also has a greater likelihood of long‐term tobacco exposure [8]. The co‐occurrence of tobacco exposure and T2DM in this population may drive a more severe disease course, heighten complication risks, and raise mortality. Notwithstanding growing awareness of the severity of this comorbidity, a paucity of systematic, contemporary assessments persists regarding the global burden of mortality and DALYs attributable to tobacco exposure among middle‐aged and older adults with T2DM. The GBD study offers a distinctive, evidence‐based framework for quantifying health loss across diverse diseases, risk factors, and geographic regions—having previously identified multiple key risk factors for diabetes, including tobacco exposure.

Assessing the global burden of mortality and DALYs associated with tobacco exposure among middle‐aged and older adults with T2DM carries critical importance for multifaceted public health rationale. First, it supports policymakers in prioritizing tobacco control measures and optimizing resource allocation. Second, it serves as a baseline for evaluating the long‐term effectiveness of public health initiatives—including tobacco taxation, smoke‐free policies, and antismoking campaigns—in alleviating the burden of T2DM in this specific population subgroup. Third, by identifying regions with the highest tobacco exposure–attributable T2DM burden, it enables the design of targeted prevention and management programs to tackle this growing public health concern. Prior studies have largely concentrated on the disease burden of T2DM linked to secondhand smoke exposure and other risk factors, with a marked absence of dedicated research focusing specifically on direct tobacco exposure and middle‐aged and older cohorts in this context [9–11]. However, tobacco exposure does not only refer to smoking but also includes secondhand smoke and chewing tobacco. Thus, the present study fills a critical gap in the existing literature and provides valuable insights into the global health impact of comprehensive tobacco exposure on middle‐aged and older adults with T2DM.

## 2. Methods

### 2.1. Study Population and Data Compilation

Leveraging contemporary epidemiological data and standardized methodologies, the GBD 2021 study quantifies health loss attributable to 371 diseases across 204 countries and territories, incorporating mortality and DALYs as core metrics [12]. This comprehensive dataset enables robust assessment of global disease burden. Specifically, our analysis centers on two key epidemiological indicators for tobacco‐attributable T2DM: mortality and DALYs. The study encompassed all 204 countries and territories, initially classified into 21 GBD regions based on geographical proximity and subsequently stratified into five SDI groups. Country inclusion within each region and corresponding counts adhere strictly to the GBD study′s official classification criteria [13, 14], with full details available on the study′s official website. Detailed methodological frameworks of the GBD 2021 study are documented in separate publications [14]. Additionally, this research strictly complied with the Guidelines for Accurate and Transparent Health Estimates Reporting (GATHER) [15].

### 2.2. Exposure Definition and GBD Variable Information

Tobacco exposure is defined as a multifaceted exposure profile that encompasses cigarette smoking, chewing tobacco use, and secondhand smoke exposure. Smoking‐related exposure includes current and former smokers: Current smokers are defined as individuals who smoke either daily or occasionally at the time of assessment, while former smokers refer to those who have refrained from all tobacco products for a minimum of 6 months. Secondhand smoke exposure is characterized by sustained contact with secondhand smoke in residential or occupational settings. Current chewing tobacco use is operationalized as consumption of such products—employing a 30‐day recall period where feasible or adhering to the nearest survey‐defined criterion—regardless of frequency (e.g., occasional, daily, or less than daily). This category includes regional formulations such as betel quid combined with tobacco [14].

Data on the association between tobacco exposure and T2DM were retrieved from the GBD 2021. Specifically, in GBD Estimate, select “Risk factor” as the category, “Tobacco” as the risk, and “Diabetes mellitus type 2” as the cause; downloadable data can then be obtained via email.

### 2.3. Data Analysis

#### 2.3.1. Overview

Data analysis commenced with a systematic assessment of the dataset structure, followed by estimation of absolute numbers and rates per 100,000 population aged ≥ 55 years (denoted as “rate/rates” for brevity) for key metrics—specifically mortality and DALYs attributable to tobacco‐attributable T2DM in adults aged 55 years and older. Analyses were conducted at global, regional, and national levels. Subsequently, temporal trends in these metrics from 1990 to 2021 were examined across regions, incorporating both absolute case counts and corresponding rates [16].

To reflect the change trends, we also calculated the estimated annual percentage change (EAPC) and relative change (RC) [16–18].

#### 2.3.2. Age–Period–Cohort (APC) Analysis of Tobacco‐Attributable T2DM Burden in Middle‐Aged and Elderly Populations

APC models disentangle and quantify the temporal dynamics of disease burden by isolating the independent effects of age, period, and birth cohort. Specifically, age effects denote the impact of physiological aging on middle‐aged and elderly individuals (≥ 55 years) with tobacco‐attributable T2DM; period effects reflect the acute influences of population‐level public health events; and cohort effects elucidate the long‐term implications of exposure factors unique to distinct birth cohorts [19]. Widely utilized in sociological and epidemiological research, APC models leverage the disentanglement of these three effects to explore the dynamic changes in disease burden [19]. APC analysis was conducted via the National Institutes of Health APC Web Tool.

#### 2.3.3. Impact of SDI on Tobacco‐Attributable T2DM Burden in Middle‐Aged and Elderly Populations

To assess the association between socioeconomic development and disease burden, Spearman correlation analysis was used to evaluate the relationship between the SDI and the burden of tobacco‐attributable T2DM [20].

#### 2.3.4. Frontier Analysis of Mortality and DALYs for Tobacco‐Attributable T2DM in Middle‐Aged and Older Adults

In this study, frontier analysis was applied to quantify the gap between observed disease burden and the theoretical minimum burden for each country/territory. This was achieved by constructing a best‐practice frontier, comprising nations with the lowest tobacco‐attributable T2DM burden at each SDI level. Unlike conventional cross‐national inequality assessments, this approach uniquely identifies countries within the same SDI quintile exhibiting disproportionately high burdens. This identification can indicate inherent structural weaknesses in healthcare systems or shortcomings in risk factor control.

Additionally, it promotes knowledge sharing among countries with comparable SDI levels by facilitating learning from frontier‐adjacent counterparts. This analytical framework dissociates economic development from disease control effectiveness, offering targeted implications for resource allocation and overcoming the limitations of traditional inequality assessments. It also has the potential to identify leading regions that can act as benchmarks for other nations. For each country and territory, an “effective difference” was computed, representing the gap between the observed disease burden and the potential burden adjusted for SDI—specifically, the disparity between the current burden of tobacco‐attributable T2DM and its SDI‐adjusted potential level [21]. The core distance metric is defined by the formula *D* = |Xobs − Xfrontier(SDI)| where *D* denotes the distance, Xobs represents the observed burden rate, and Xfrontier(SDI) stands for the SDI‐matched frontier benchmark. The unit of *D* is consistent with that of the burden indicator (e.g., DALYs per 100,000 person‐years) [21].

#### 2.3.5. Projection of Tobacco‐Attributable T2DM Burden in Middle‐Aged and Older Adults

To project future trends in tobacco‐attributable T2DM burden, the Bayesian age–period–cohort (BAPC) model was implemented. This statistical framework is designed for analyzing and projecting age, period, and birth cohort effects on population health outcomes, including mortality and disease incidence rates. Critically, by harnessing Bayesian approaches, the model effectively accommodates complex data structures and quantifies inherent uncertainties in population‐level analyses.

The BAPC model decomposes population‐level data into three distinct, interpretable components: (1) Age effect refers to the inherent traits or risk profiles associated with different age groups, illustrating how an individual′s risk or incidence of the target outcome changes as they age. (2) Period effect captures time‐specific factors affecting all age groups simultaneously, typically reflecting temporal shifts in external variables—including social, economic, and environmental conditions—across different timeframes. (3) Cohort effect refers to shared characteristics or risk trajectories among individuals born in the same year, capturing unique exposures and influences that a specific birth cohort encounters throughout its lifespan. Detailed methodological specifications are available in the supporting information [22].

## 3. Results

### 3.1. Global and Regional Trends in Tobacco Exposure–Attributable T2DM Burden Among Middle‐Aged and Older Adults

The GBD 2021 database provided complete coverage for all 204 countries and territories, confirming consistency between the number of countries/territories included in the five SDI quintiles and 21 GBD regions and their intrinsic regional classifications. In 2021, global mortality attributable to tobacco exposure among adults aged ≥ 55 years was 140,767.54 (95% uncertainty interval [UI]: 88,450.01–194,600.29), corresponding to a mortality rate of 9.47 per 100,000 population (95% UI: 5.95–13.10). By SDI quintile, low–middle‐SDI regions recorded the highest mortality rate (16.06 per 100,000 population, 95% UI: 9.70–22.43), while high‐SDI regions had the lowest (4.77 per 100,000 population, 95% UI: 3.21–6.53). At the regional level, Oceania exhibited the highest mortality rate (57.60 per 100,000 population, 95% UI: 32.44–83.12), followed by Southern Sub‐Saharan Africa (29.75 per 100,000 population, 95% UI: 17.29–42.09). Southeast Asia (17.93 per 100,000 population, 95% UI: 11.06–24.91) and South Asia (14.31 per 100,000 population, 95% UI: 8.50–20.56) followed, while High‐Income Asia Pacific (2.57 per 100,000 population, 95% UI: 1.70–3.58) and Australasia (2.95 per 100,000 population, 95% UI: 1.81–4.24) recorded the lowest rates. Globally, the EAPC in mortality rate was −0.48 (95% confidence interval [CI]: −0.58 to −0.39), indicating a modest but significant decline. However, substantial heterogeneity existed across SDI quintiles and regions: High‐SDI regions showed the steepest decline (EAPC: −2.27, 95% CI: −2.51 to −2.03), while low–middle‐SDI regions exhibited an upward trend (EAPC: 0.34, 95% CI: 0.30 to 0.38). Regionally, High‐Income Asia Pacific (EAPC: −3.38, 95% CI: −3.57 to −3.12) and Western Europe (EAPC: −2.49, 95% CI: −2.62 to −2.36) had the most pronounced declines, whereas Central Asia (EAPC: 1.41, 95% CI: 0.81–2.01) and Eastern Europe (EAPC: 1.91, 95% CI: 0.73–3.12) showed increasing trends. Gender disparities persisted (male mortality rates 1.5–2.5× higher than female rates), but EAPC results indicated declining mortality burden for both sexes (Table S1).

For DALYs, global burden attributable to tobacco exposure among adults aged ≥ 55 years was 5,934,138.22 (95% UI: 3,798,274.69–8,469,349.66), corresponding to a rate of 399.34 per 100,000 population (95% UI: 255.61–569.95). Significant regional disparities existed: Middle‐SDI quintiles had the highest DALYs rate (439.15 per 100,000 population, 95% UI: 272.92–623.69), while high‐SDI quintiles had the lowest (298.16 per 100,000 population, 95% UI: 199.12–424.48). Regionally, Oceania (1888.54 per 100,000 population, 95% UI: 1113.66–2702.64) and Southern Sub‐Saharan Africa (880.68 per 100,000 population, 95% UI: 525.42–1245.94) had the highest rates, followed by North Africa and the Middle East (692.00 per 100,000 population, 95% UI: 406.02–1005.06). Western Europe (206.46 per 100,000 population, 95% UI: 137.70–296.73) and Australasia (152.66 per 100,000 population, 95% UI: 97.70–222.40) recorded the lowest rates. Globally, the DALYs rate showed a slight upward trend (EAPC = 0.08, 95% CI: 0.01–0.15), with low–middle‐SDI quintiles exhibiting the strongest increase (EAPC = 0.61, 95% CI: 0.58–0.65), while high‐SDI quintiles (EAPC = −0.38, 95% CI: −0.51 to −0.25) and middle‐SDI quintiles (EAPC = −0.28, 95% CI: −0.36 to −0.19) showed declines. Regionally, Central Asia had the steepest upward trend (EAPC = 1.90, 95% CI: 1.52–2.28). Notably, while female DALYs burden decreased consistently over 1990–2021, male burden continued to rise (Table S2).

Figure 1 presents 2021 mortality and DALYs counts and rates for tobacco exposure–attributable T2DM burden among middle‐aged and older adults across 204 countries/territories, alongside EAPC trends from 1990 to 2021.

Figure 1The number of (a) deaths and (b) DALYs from T2DM attributable to tobacco exposure in 2021. The rate per 100,000 aged ≥ 55 population of (c) deaths and (d) DALYs from T2DM attributable to tobacco exposure in 2021. The EAPC of (e) deaths and (f) DALYs from T2DM attributable to tobacco exposure from 1990 to 2021.

**Figure:**
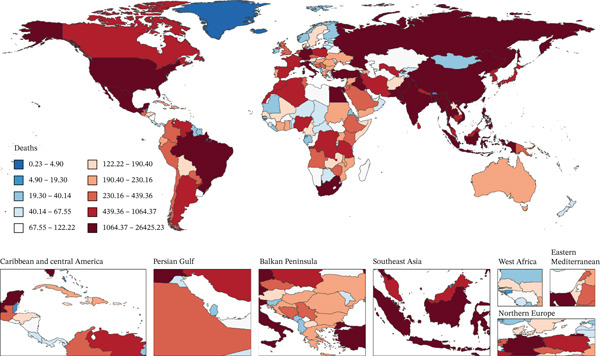
(a)

**Figure:**
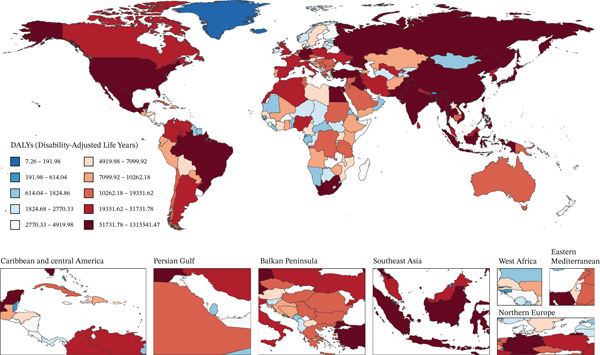
(b)

**Figure:**
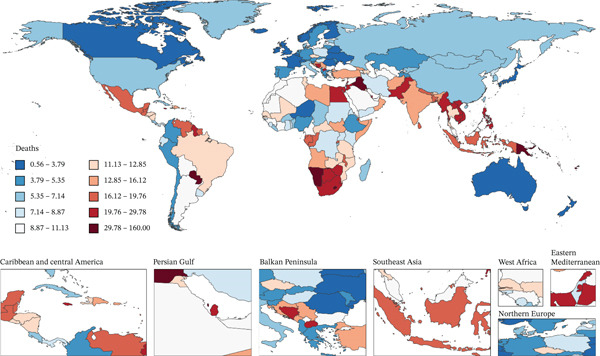
(c)

**Figure:**
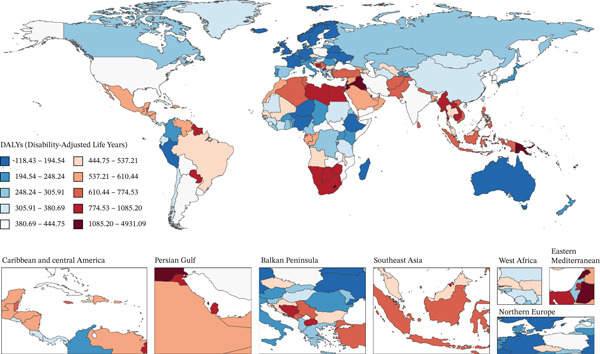
(d)

**Figure:**
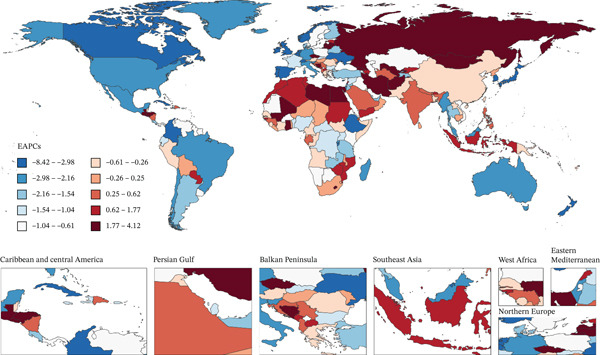
(e)

**Figure:**
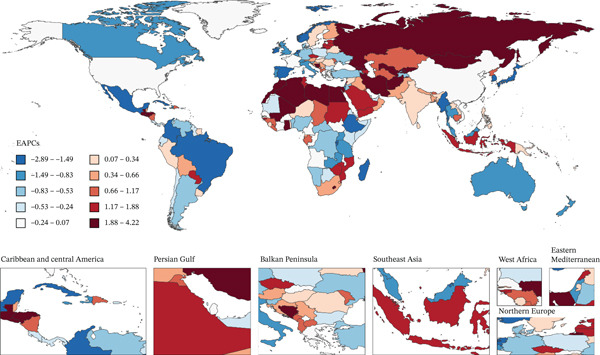
(f)

### 3.2. Temporal, Gender, and Age‐Specific Trends in Tobacco Exposure–Attributable T2DM Among Middle‐Aged and Older Adults

**Figure 2:**
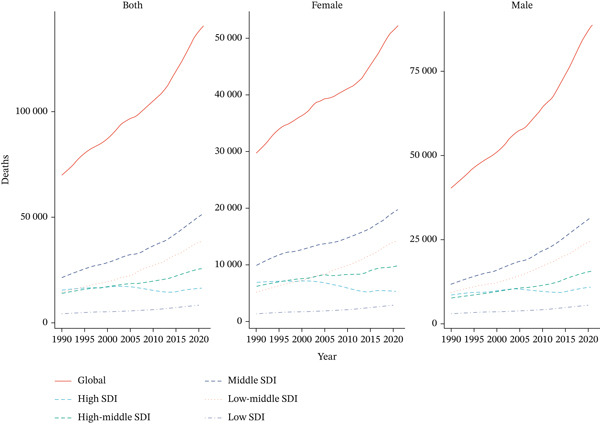
The trends of number in deaths and DALYs from T2DM attributable to tobacco exposure categorized by global and five SDI regions from 1990 to 2021.

Over the past three decades, the number of deaths and DALYs attributed to T2DM due to tobacco exposure has increased annually among middle‐aged and elderly individuals worldwide. Notably, around 2002, the number of deaths in high‐SDI regions began to decline. This downward trend was observed in both genders, and thereafter, the death count in high‐SDI regions remained lower than that in high–middle and low–middle‐SDI regions. During the same period, the overall growth rate of DALYs in males also slowed down, which was lower than the growth rates in high–middle and low–middle‐SDI regions (Figure 2 and Figure S1). However, globally, the mortality rate of T2DM caused by tobacco exposure has fluctuated over time, despite a slight overall decrease. Nevertheless, for males in low–middle‐SDI regions and the total DALYs, this rate continued to increase (Figure S2 and Figure S3).

Distinct patterns in mortality counts and rates attributable to tobacco exposure were observed across age groups and sexes. Both mortality counts and rates increased with age, with the highest burden observed in older adults. Mortality counts peaked at 15,405.29 (95% UI: 10,941.77–20,549.63) in males aged 70–74 years and at 7100.28 (95% UI: 3229.52–11,092.50) in females aged 80–84 years. Corresponding mortality rates peaked at 46.77 per 100,000 population (95% UI: 29.60–66.27) in males aged 90–94 years and at 18.00 per 100,000 population (95% UI: 8.47–28.45) in females aged 85–89 years. Males consistently exhibited higher mortality counts and rates than females, with the oldest age groups carrying the highest burden (Figure 3a).

Figure 3The trends of case number and rate per 100,000 aged ≥ 55 population in deaths and DALYs from T2DM attributable to tobacco exposure across different genders, female and male, by age groups in global and five SDI regions. The shaded area represents the corresponding 95% UI.

**Figure:**
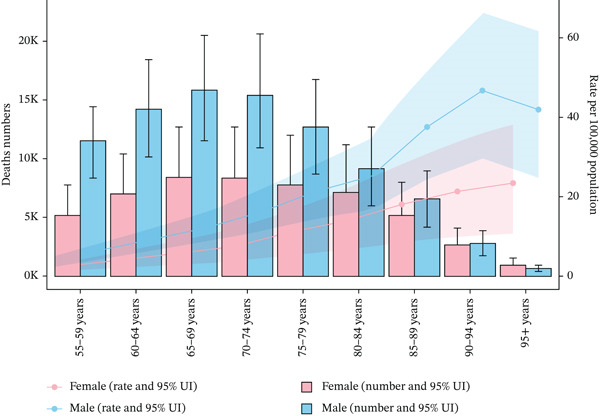
(a) Deaths

**Figure:**
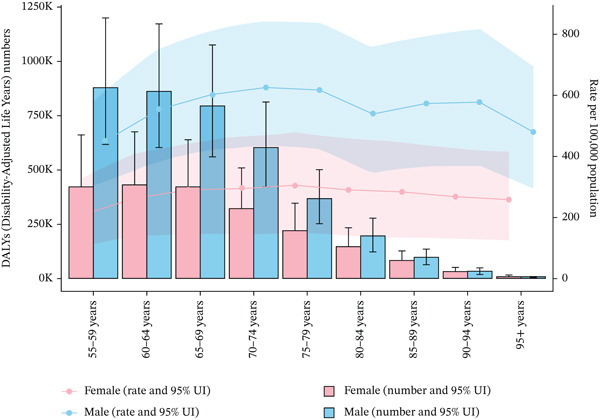
(b) DALYs

For DALYs, DALYs counts peaked at 879,146.94 (95% UI: 616,855.77–1,197,236.59) in males aged 55–59 years, while females peaked at 10,179.13 (95% UI: 4956.07–16,384.47) in the 95+ years age group. DALYs rates were highest in males aged 70–74 years (625.86 per 100,000 population, 95% UI: 434.96–843.08) and females aged 65–69 years (290.76 per 100,000 population, 95% UI: 148.31–443.98). In general, males carried a higher DALYs burden in the middle‐aged group (55–74 years), while females showed a more prominent DALYs burden in the elderly group (≥ 65 years) (Figure 3b).

### 3.3. APC Analysis of Tobacco Exposure–Attributable T2DM Burden Among Middle‐Aged and Older Adult

APC analysis revealed key patterns in tobacco exposure–attributable T2DM burden. Figure 4a shows that mortality rates increased with advancing age across all time periods, with a steeper rise observed in older adults (75–80 years). This indicates heightened mortality risk in the oldest age groups. Figure 4b demonstrates that earlier birth cohorts (e.g., born ~1890) exhibited higher mortality rates, while later cohorts (e.g., born 1940–1950) had lower rates. Figure 4c indicates a gradual decline in mortality rates across all age groups over time.

Figure 4Results of age–period–cohort analysis for deaths: trends and deviations in rates per 100,000 aged ≥ 55 population across (a) age groups, (b) birth cohort, and (c) year dimensions.

**Figure:**
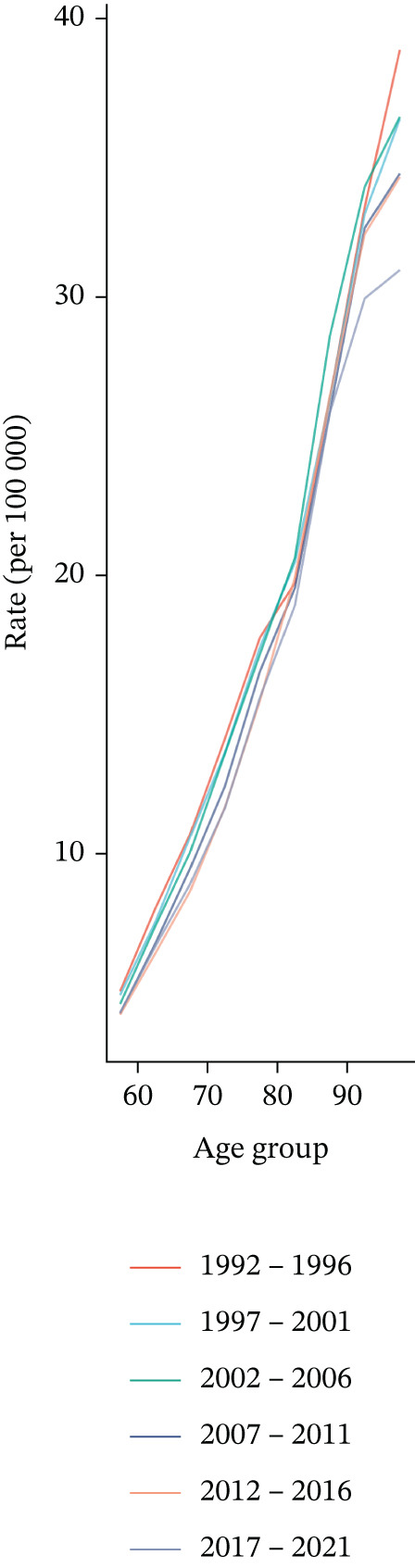
(a)

**Figure:**
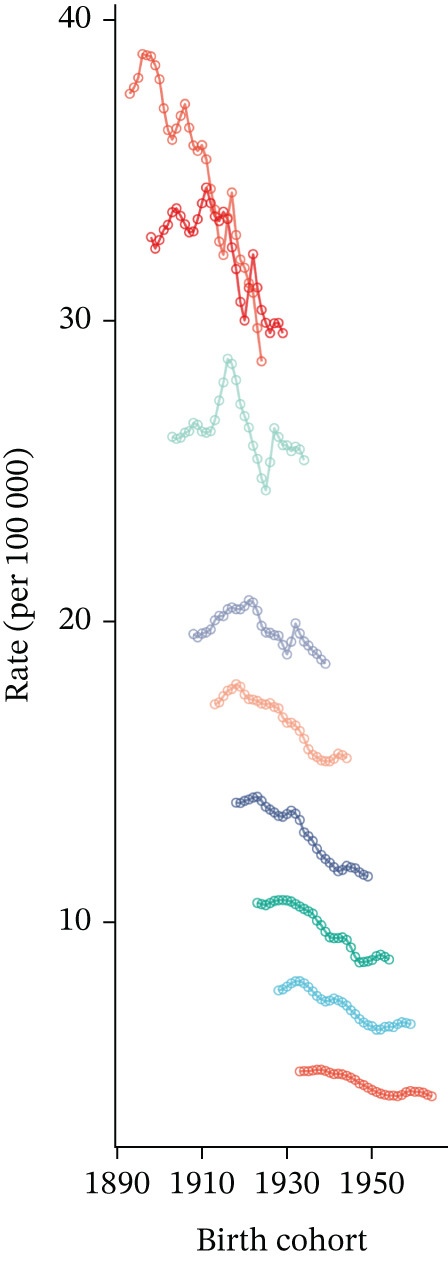
(b)

**Figure:**
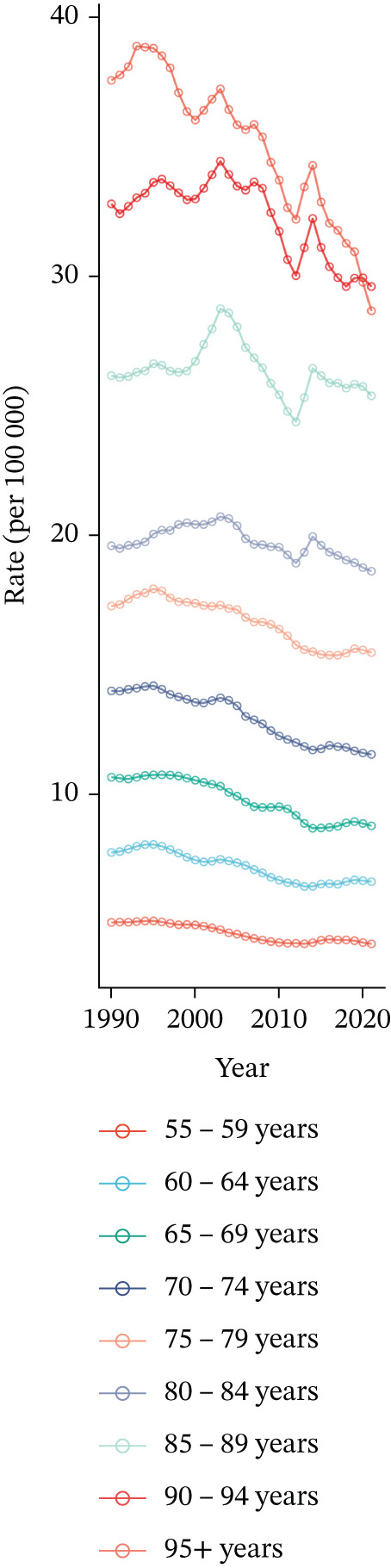
(c)

Data from Figure S5A presents the age‐specific DALYs rates of T2DM across six 5‐year periods, ranging from 1992–1996 to 2017–2021. Overall, the DALYs rate exhibited an inverted U‐shaped distribution with age: It shows that the peak was observed in the 70–74 years, followed by a gradual decrease. Over time, the DALYs rate in elderly age groups (e.g., 80–84 years and above) showed slight fluctuations and tended to stabilize or decrease slightly in recent periods. In contrast, the DALYs rate in younger age groups (e.g., 55–59 years) remained relatively stable. Period‐specific trends indicated that the burden of T2DM was concentrated in 55+ years populations, with no significant increase observed over decades. Cohort‐specific analysis of Figure S5B revealed the longitudinal trend of DALYs rates among birth cohorts born between 1893 and 1964. Across all age groups, later‐born cohorts (e.g., 1950–1964) had generally stable or slightly increased DALYs rates at younger ages (55–59 years). For elderly age groups (e.g., 70–74 years), the DALYs rate peaked in earlier cohorts and then stabilized or decreased in subsequent cohorts. Notably, the oldest cohorts (e.g., those aged ≥ 95 years, corresponding to the 1893–1900 birth cohort) exhibited a distinct downward trend. Figure S5C shows the following trends in mortality rate by age group. Younger age groups (e.g., 55–59 years, 60–64 years): The mortality rate showed an upward trend after 2010. Elderly age groups (e.g., 90–94 years, ≥ 95 years): The mortality rate was relatively high in the early stage but exhibited significant fluctuations or changes in the later stage.

### 3.4. Results of the Relationship Between T2DM Attributable to Tobacco Exposure in Middle‐Aged and Elderly Patient Burden and SDI

Spearman correlation analysis revealed a statistically significant negative correlation between the T2DM mortality rate and SDI (*r* = −0.3445, 95% CI: −0.4671 to −0.2072, *p* = 5.343 × 10^−7^). Notably, the disease burden was highest in middle‐SDI regions, whereas it was relatively lower in low or high‐SDI regions. For instance, pacific island countries with low SDI—such as Kiribati, Fiji, and Nauru—exhibited significantly higher mortality rates than high‐SDI countries including Singapore, Japan, and Switzerland (Figure S5A). Consistently, the results for DALYs were nearly consistent with those for mortality, as shown in Figure S5B.

### 3.5. Results of the Frontier Analysis

From the frontier analysis results, this study evaluated the performance of mortality rates from T2DM among patients aged 55 years and older across 204 regions in 2021. Regions with the largest efficiency differences: The 15 regions with the most significant efficiency differences were primarily distributed in Oceania and Africa, including Kiribati, Fiji, and Nauru. The actual mortality rates in these regions were substantially higher than the theoretical optimal value (frontier line) corresponding to their SDI levels.

Regions with the smallest efficiency differences in low‐SDI countries (SDI < 0.5) included Somalia, Niger, Ethiopia, Chad, and Burundi. The actual mortality rates in these countries were close to or equal to the theoretical optimal value, indicating that despite limited resources, their healthcare systems have achieved or approached the potential optimal performance in T2DM management at their current development level.

Regions with the smallest efficiency differences in high‐SDI countries included Taiwan Province of China, Denmark, the Republic of Korea, the United States, and Austria. The effective differences in these high‐income regions were relatively small (Figure 5).

Figure 5Frontier analysis, represented by the solid black lines, explores the relationship between SDI and rate per 100,000 aged ≥ 55 population for deaths in the context of T2DM attributable to tobacco exposure in middle‐aged and elderly patients. The color gradient in (a) illustrates the progression of years, ranging from light shades representing 1990 to the darkest shades denoting 2021. In (b), each dot signifies a specific country or territory for the year 2021, with the top 15 countries displaying the most significant deviation from the frontier labeled in black. Countries with low SDI (> 0.455) and minimal deviation from the frontier are highlighted in blue, while those with high SDI (> 0.805) and notable deviation for their developmental level are emphasized in red. The direction of change from 1990 to 2021 in rate per 100,000 aged ≥ 55 population is indicated by the color of the dots: Red dots represent a decrease, while blue dots signify an increase. The larger distance means worse performance relative to the frontier.

**Figure:**
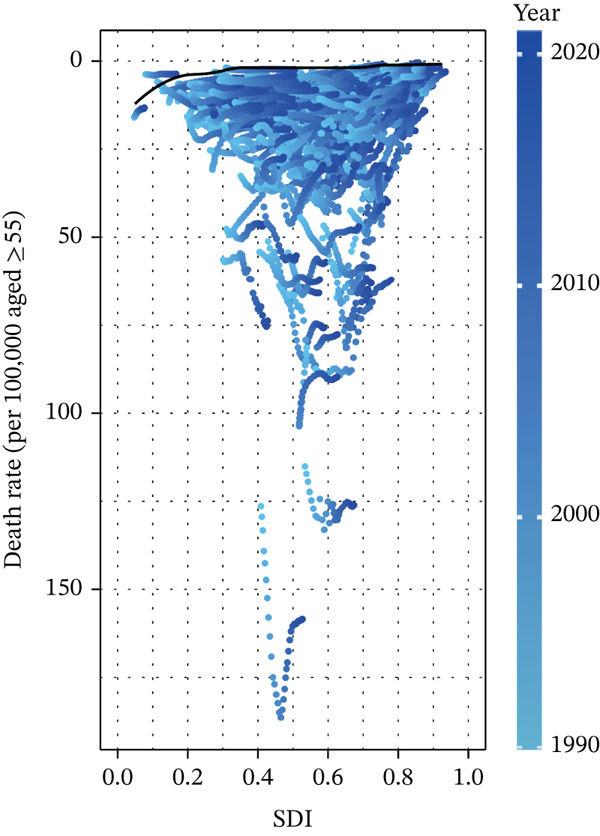
(a)

**Figure:**
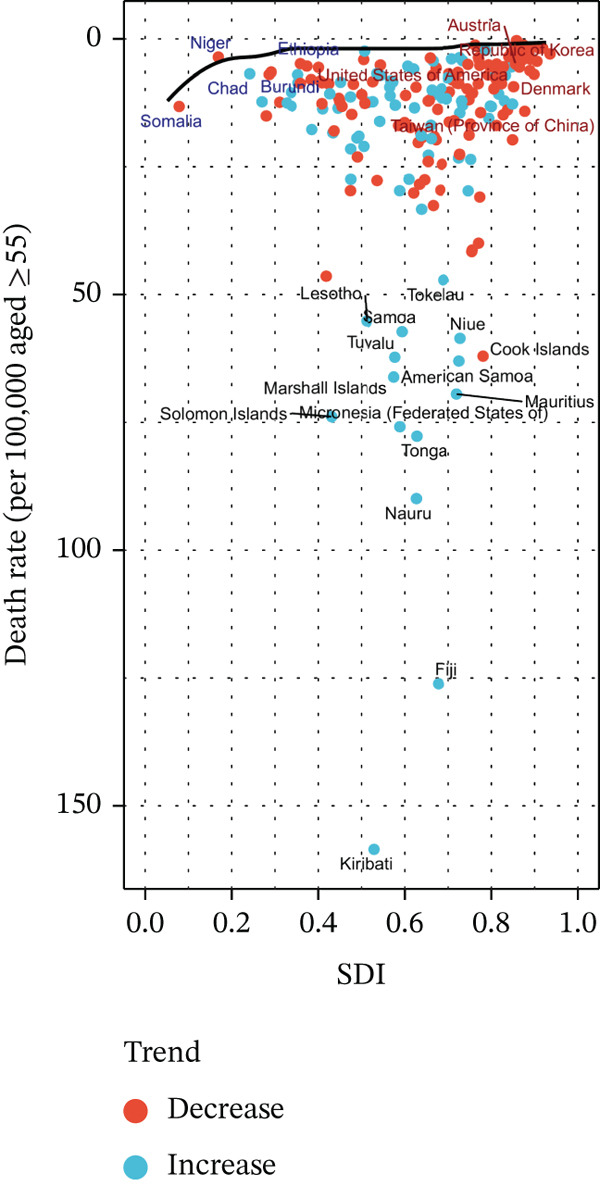
(b)

Consistently, Figure S6 shows the results for DALYs were nearly consistent with those for mortality.

### 3.6. Results of the Predictive Analysis

Figure 6 presents the projected death rate of T2DM attributed to tobacco exposure in 55+ years patients from 2022 to 2042. Regarding mortality rate, projections indicated that the overall mortality rate would continue to decrease in both genders in the future: It was projected to be 9.25 per 100,000 population in 2030 (95% CI: 8.64–9.85) and 8.57 per 100,000 population in 2042 (95% CI: 6.89–10.24) (Figure 6a). Subgroup analysis by gender revealed the following trends: The male mortality rate was likely to remain stable in 2030, at 13.27 per 100,000 population (95% CI: 12.51–14.03), and was projected to be 12.53 per 100,000 population in 2042 (95% CI: 10.31–14.75) (Figure 6b), whereas the female mortality rate was projected to decrease in the future, with values of 5.98 per 100,000 population in 2030 (95% CI: 5.52–6.44) and 5.33 per 100,000 population in 2042 (95% CI: 4.20–6.45) (Figure 6c and Table S3).

Figure 6Predicted trends of T2DM attributable to tobacco exposure over the next 15 years (2022–2042): (a) rate per 100,000 aged ≥ 55 population of deaths of both; (b) rate per 100,000 aged ≥ 55 population of deaths of male; (c) rate per 100,000 aged ≥ 55 population of deaths of female. Dark blue lines represent the true trend during 1990–2021; black lines and dark blue shaded regions represent the predicted trend and its 95% CI.

**Figure:**
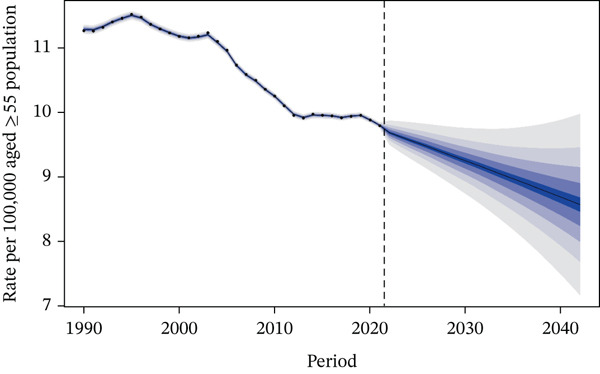
(a) Both sex

**Figure:**
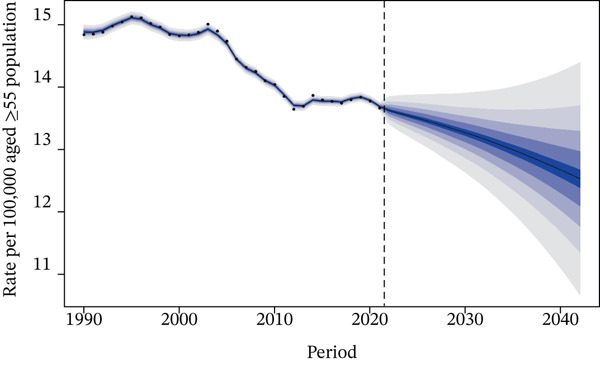
(b) Male

**Figure:**
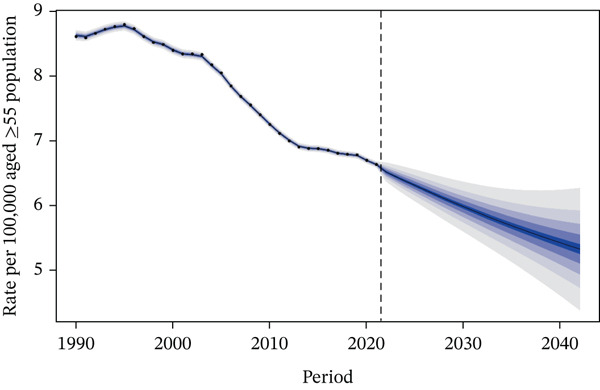
(c) Female

For the DALYs rate, a continuous upward trend was projected in the future, both in the overall population and in gender‐specific subgroups (Figure S7 and Table S4).

## 4. Discussion

### 4.1. Discussion on Trend and Disparities

#### 4.1.1. Global and Regional Trends

From 1990 to 2021, the global number of deaths from T2DM attributed to tobacco exposure increased by 101.10%, while the DALYs increased by 140.38%. This indicates that the impact of tobacco exposure on T2DM‐related health outcomes remains persistent and is growing. However, the mortality rate exhibited a downward trend. It indicates that although the absolute number of cases is on the rise owing to population growth and aging, individual‐level risk may have stabilized or decreased in some contexts. This phenomenon could be attributed to improved healthcare access, better diabetes management in certain regions, and tobacco cessation programs—factors that may have contributed to a reduction in incidence [23]. Notably, this finding highlights the population‐level effectiveness of these interventions. Nevertheless, the overall increase in the number of deaths underscores the need for additional efforts.

#### 4.1.2. Disparities Across SDI Quintiles

During the entire study period, low‐SDI regions consistently had the lowest number of cases related to tobacco exposure–induced T2DM. This observation may be attributed to either a lower prevalence of diabetes itself or the less prominent impacts of tobacco exposure–associated complications [3, 24]. However, this does not imply favorable health outcomes, as cotrending factors such as limited healthcare infrastructure, high poverty‐related health literacy gaps, and competing disease burdens may constrain improvements in these regions. However, middle‐SDI regions exhibited the heaviest disease burden in terms of absolute counts. This observation may be attributed to three key factors: relatively high smoking rates, rising diabetes incidence amid ongoing socioeconomic transitions in these regions [25], and potentially less effective healthcare systems for addressing the complex interaction between tobacco exposure and T2DM [26].

Notably, the most severe burden rates were observed in low–middle‐SDI regions in both 1990 and 2021. This pattern underscores the vulnerability of these regions, likely stemming from limited healthcare resources, inadequate awareness of the tobacco‐diabetes interplay, and socioeconomic factors that amplify associated risks. Furthermore, the substantial relative increases in case counts and burden rates in low–middle‐SDI regions, along with the highest EAPC for DALYs, thus underscore an urgent need for targeted interventions in these regions. Conversely, while low‐SDI regions still maintained the lowest case counts, their relatively slow rates of amelioration indicate unique challenges, potentially including healthcare access barriers and poverty‐related gaps in health education.

Conversely, high‐SDI regions demonstrated the most marked reduction in burden rates, accompanied by the lowest EAPC values for both deaths and DALYs. This trend aligns with ecological associations observed in model analyses, including stronger public health policies (e.g., tobacco taxation, smoke‐free laws), improved healthcare access, scaled‐up smoking cessation programs, and greater population awareness of tobacco exposure–T2DM risks. However, this decline is not attributable to these factors in isolation: cotrending improvements such as stabilized BMI growth, widespread adoption of healthy dietary patterns, expanded diabetes screening coverage, and advances in T2DM pharmacotherapy [27, 28].

#### 4.1.3. Regional Variations

Among the 21 GBD regions, South Asia registered the highest absolute numbers of deaths and DALYs associated with the outcome in 2021. Key contributors to this pattern likely include the region′s large population size, high smoking prevalence, and a growing T2DM burden [29, 30]. In contrast, Oceania showed the highest rates for both deaths and DALYs. This observation may stem from region‐specific factors such as genetic predisposing factors and lifestyle‐related influences (e.g., high obesity prevalence, traditional dietary shifts toward energy‐dense foods) [31, 32].

Eastern Europe exhibited the highest EAPC values for both deaths and DALYs, signaling a worsening trend that warrants in‐depth exploration of local smoking patterns, diabetes management protocols, and socioeconomic contexts. In contrast, High‐Income Asia Pacific and Central Latin America had the lowest EAPC values for deaths and DALYs, respectively—this pattern further underscores the positive effects of robust healthcare systems and effective tobacco control interventions [33].

Further analysis across 204 countries indicates that the observed disparities may be attributed to factors such as trade policies influencing tobacco supply, regional cultural norms regarding smoking, and variations in diabetes screening practices across different regions. For example, the effectiveness of policy implementation varies with regional contexts. Although Gulf countries have generally adopted demand‐side measures under the Framework Convention on Tobacco Control (FCTC), progress in price policies and illicit trade control lags behind [34]. Media analyses in Australia have revealed that “nanny state” rhetoric and analogies to prohibition are frequently employed to oppose endgame policies [35]. In contrast, the Philippines has stabilized its illicit tobacco market share at 16% through successive tax increases, which did not grow alongside rising tax rates [11]. Additionally, studies in Lithuania have identified state‐owned tobacco factories in neighboring Belarus as primary sources of illicit tobacco, necessitating targeted border control measures [36].

Furthermore, diabetes screening coverage exhibits significant disparities across different regions. In Saudi Arabia, 52.9% of individuals with diabetes demonstrate adequate awareness of screening programs, with screening rates in rural areas being lower than those in urban regions [37, 38]. Similarly, in British Columbia, Canada, the screening rate increased from 88% in 2004 to 96% in 2019; however, the proportion of screenings utilizing the one‐step approach fluctuated considerably due to changes in clinical guidelines [39]. In contrast, screening rates in African regions are generally low due to resource constraints, with basic examinations predominantly relying on nonprofessional personnel [40]. These disparities underscore the critical role of economic development and healthcare infrastructure in the implementation of screening initiatives.

#### 4.1.4. Gender Differences

Across the entire study period, males consistently exhibited higher death counts, DALYs, and burden rates than females. This observation is consistent with previous findings indicating that males have higher smoking prevalence and may be more vulnerable to health complications associated with tobacco exposure. Notably, females showed slower growth in case counts and more pronounced reductions in burden rates—outcomes that may stem from gender‐specific differences in smoking cessation rates, healthcare‐seeking behaviors, or biological differences that modulate the progression of tobacco‐attributable T2DM [24, 41].

#### 4.1.5. Implications and Future Directions

The present findings underscore the necessity of region‐ and population‐specific interventions to alleviate the burden of tobacco‐attributable T2DM among middle‐aged and elderly individuals. Notably, for low–middle‐SDI regions, priority measures should include strengthening healthcare infrastructure, expanding smoking cessation programs, and improving awareness of tobacco exposure–T2DM association. In contrast, high‐SDI regions should sustain efforts to reinforce public health policies and enhance surveillance systems, thereby maintaining the declining trend in disease burden [41].

Beyond intervention strategies, additional research is warranted to investigate the underlying mechanisms driving these disparities, including socioeconomic, cultural, and biological determinants [32] Additionally, evaluating the efficacy of targeted interventions across diverse settings is critical for formulating evidence‐based strategies to mitigate the global impact of tobacco exposure on T2DM. Future studies could also explore in greater depth the interactions among tobacco exposure, T2DM, and other comorbidities, which would foster a more holistic understanding of this complex public health issue.

### 4.2. Synthesis of Gender and Age Trends

Males consistently demonstrate higher absolute counts and rates of tobacco exposure–attributable T2DM mortality compared to females. This gender disparity can be largely driven by higher smoking prevalence among males, as well as their potential greater vulnerability to tobacco exposure–related health complications [42]. For absolute mortality, the age‐related pattern—peaking at 65–69 years and declining after 95+ years—may stem from two key mechanisms: cohort effects (where individuals aged 65–69 likely experienced higher tobacco exposure during their lifetime) and survival effects (where fewer individuals survive beyond 95 years due to cumulative health risks). Additionally, the male mortality rate increases from 55 to 59 years, peaks at 90–94 years, and then decreases; in contrast to the sustained increase in the female rate, this difference may reflect gender‐specific variations in smoking behaviors, T2DM progression trajectories, and healthcare engagement.

In terms of DALYs, both sexes exhibit a predominantly declining trend in DALYs counts starting at 55–59 years, while DALYs rates rise until 65–74 years and then decrease. This pattern points to a complex interplay between disease onset, progression dynamics, and clinical management across the lifespan. It may be influenced by improved T2DM management and smoking cessation interventions in middle age, which could reduce subsequent disease burden. However, the upward trend in DALYs rates until 65–74 years indicates that individuals in this age group remain vulnerable to tobacco exposure–attributable T2DM complications, despite the overall declining burden [43].

These findings underscore the importance of implementing targeted public health strategies. For males, given their higher burden of tobacco exposure–attributable T2DM mortality, scaled‐up smoking cessation interventions and targeted T2DM screening efforts are paramount. For females, the sustained upward trend in rates highlights the necessity of exploring and addressing sex‐specific factors that may modulate their risk of tobacco exposure–related T2DM. Females are more likely to be affected primarily due to exposure to secondhand smoke [44]. With respect to age‐specific patterns, middle‐aged populations (55–59 years) could derive benefit from preventive measures aimed at reducing T2DM onset and its complications, while older adults (65–74 years) may require more focused management of existing conditions to mitigate disability and premature mortality.

Further investigations are warranted to unravel the mechanistic drivers of these sex‐ and age‐specific disparities. This includes exploring biological susceptibilities that may render males more prone to tobacco exposure–related T2DM complications, as well as sociobehavioral determinants influencing smoking patterns and healthcare utilization across different age and sex subgroups.

### 4.3. APC Insights Into T2DM Attributable to Tobacco Exposure in Middle‐Aged and Elderly Patients

The mortality rate gradually increased starting from 55 years of age, with a more pronounced rise observed between 75 and 80 years. This observation aligns with the well‐established consensus that “aging is a primary risk factor for T2DM and its complications.” With advancing age, the human body undergoes physiological changes—such as decreased insulin sensitivity, impaired *β* cell function, and accumulation of comorbidities—which increase an individual′s susceptibility to the adverse effects of tobacco exposure–induced T2DM [45, 46]. Furthermore, the elderly population has longer cumulative tobacco exposure over their lifetime, which may contribute to a progressive increase in their mortality risk. This phenomenon underscores the importance of long‐term tobacco cessation interventions and T2DM management strategies targeting middle‐aged and elderly populations [47].

The impact of cohort/period effects became more prominent with increasing age, as evidenced by the declining curves in Figure 4c. These observations indicate that historical factors and intergenerational trends play a critical role in shaping the mortality burden of tobacco exposure–related T2DM. For instance, variations in smoking prevalence across generations, accessibility of tobacco products, and shifts in smoking‐related social norms may have exerted significant influences on the level of tobacco exposure in each cohort [48, 49].

The downward trend in mortality rate over time and the reduction in period‐specific relative risk are positive signals. These trends may reflect the effectiveness of global tobacco control measures, advancements in T2DM prevention and treatment technologies, and improved public health awareness. Policies such as tobacco taxation, implementation of smoke‐free regulations, and antismoking campaigns may have helped reduce population‐level tobacco exposure, thereby alleviating the disease burden of tobacco exposure–related T2DM [42, 50]. However, the mortality burden of tobacco exposure–related T2DM remains nonnegligible, indicating substantial room for improvement in these interventions.

The finding that younger cohorts had lower mortality rates is consistent with the hypothesis that “recent generations are more likely to benefit from evolving public health initiatives and changing social attitudes toward smoking” [51]. Younger individuals may be more receptive to health education, have better access to smoking cessation resources, and possess higher awareness of the risks associated with tobacco exposure and T2DM [52]. This phenomenon highlights the importance of sustained health promotion efforts to ensure the continuation of these positive trends in future generations.

Fluctuations in mortality rates were observed among early cohorts between 1995 and 2010. This phenomenon suggests that specific historical events, policy changes, or socioeconomic factors during these periods exerted profound impacts on the mortality trends of tobacco exposure–related T2DM. For example, in the 1990s, the US tobacco industry engaged in lobbying activities at the state and local government levels [53]; in May 2003, the World Health Organization (WHO) released the FCTC, which was subsequently signed and implemented by many countries—with the effectiveness of tobacco control gradually becoming evident in subsequent years [54, 55]. Previous studies investigating tobacco exposure burden in the ASEAN region have indicated that the tobacco epidemic can be broadly categorized into four distinct stages: the initial phase, featuring low prevalence levels; the second phase, defined by a rising prevalence that shows no obvious downward tendency; the third phase, in which downward trends start to appear while prevalence still stays at a high level; and the fourth phase, where prevalence has dropped to a relatively low range. This phenomenon bears a close connection to national tobacco control policies geared toward adolescent populations in various countries [56]. Thailand stands out as a typical case: Despite upholding progressive positions in other tobacco control areas, it did not introduce its inaugural tobacco control laws tailored to young people or launch school‐oriented intervention programs until 2017 [57]. In light of adolescents′ elevated susceptibility to advertising exposure, such delays exert notable ramifications [58].

In general, the findings of the APC analysis indicate that considerable strides have been achieved in managing the global burden of T2DM induced by tobacco exposure. It is worth noting that the elderly population continues to be the primary target of prevention and control initiatives, highlighting the need to strengthen the comprehensive management of diabetes and tobacco exposure in this group. Furthermore, the cohort effect indicates that early interventions targeting tobacco exposure prevention yield long‐term benefits, which offers an evidence‐based foundation for optimizing resource allocation and developing targeted prevention and control strategies. Meanwhile, the stability of period‐specific trends may reflect global efforts in diabetes and tobacco control, whereas the decline in cohort‐specific outcomes underscores the long‐term benefits brought about by advances in healthcare and tobacco control policies.

### 4.4. Global Disparities

Middle and high–middle‐SDI regions demonstrate the most substantial tobacco exposure–related T2DM burden, while high‐SDI and low‐SDI regions exhibit significantly lower burden levels. This pattern highlights the vital role of socioeconomic and demographic factors in shaping the global distribution of this burden.

Notably, middle‐SDI and high–middle‐SDI regions are typically marked by rapid urbanization and industrialization, which drive lifestyle shifts including increased sedentary behavior, unhealthy dietary patterns, and elevated stress levels [59]. These shifts can elevate tobacco exposure rates and T2DM incidence. Additionally, healthcare systems in these regions often lack the capacity to adequately address the complex interplay between tobacco exposure and T2DM, ultimately leading to suboptimal health outcomes [9].

In contrast, high‐SDI regions generally possess robust healthcare infrastructure, enhanced access to preventive services, and more effective tobacco control policies—factors that likely account for their lower burden [60]. Conversely, low‐SDI regions may exhibit lower absolute case counts due to younger demographic structures and reduced prevalence of risk factors such as tobacco exposure and obesity [61]. However, this does not equate to superior health outcomes, as these regions confront distinct challenges, including limited healthcare resources.

Accordingly, interventions in middle‐SDI regions need to be adapted to resource constraints: Smoking cessation interventions should be integrated into routine diabetes follow‐up procedures. Experience from high‐SDI and well‐controlled regions, such as culturally tailored and community resource‐integrated mail‐based interventions (e.g., the Koori Quit Pack for smoking cessation and chatbots, remote monitoring, and social support), can serve as reference models [11, 62–65]. Meanwhile, the ≥ 75‐year‐old T2DM population exhibits significant mortality risk and thus should be included in core intervention. This approach helps correct the cognitive bias that “smoking cessation in the elderly is meaningless.”

### 4.5. Prevention and Control Strategies

Frontier analysis effectively evaluates how healthcare systems across 204 regions translate sociodemographic resources (measured by the SDI value) into health outcomes for 55+ years individuals with tobacco exposure–induced T2DM.

Regions with the largest efficiency differences—primarily distributed in Oceania and Africa (e.g., Kiribati and Fiji)—exhibited actual mortality rates that far exceeded the theoretical optimal values corresponding to their SDI levels. This phenomenon may be attributed to inadequate healthcare access in these island nations [66], as well as the impact of high obesity prevalence on T2DM‐related mortality rates and life expectancy [11].

By contrast, low‐SDI regions such as Somalia and Niger showed minimal efficiency gaps, with actual rates approaching the optimal values. Despite limited resources, these regions demonstrated resource efficiency through targeted interventions. High‐SDI regions—including Taiwan Province of China, Denmark, and the Republic of Korea—also exhibited small efficiency gaps, driven by robust tobacco control measures, mature T2DM management systems, and high levels of health literacy [67–69].

Consistent with the mortality findings, results for DALYs further confirmed the impact of tobacco exposure on both survival and incidence outcomes. These insights suggest that regions with large efficiency gaps require urgent investment in tobacco control and T2DM care infrastructure; the high‐efficiency models of low‐SDI regions merit investigation into their scalability; and high‐SDI regions can advance precision public health while supporting global capacity building. Future studies are needed to refine region‐specific strategies for addressing tobacco exposure–induced T2DM.

In the context of policy benchmarking, the findings of this study provide a precise framework for cross‐national policy alignment. Specifically, countries with deviated disease burdens in the middle‐SDI category can draw on experiences from high‐performing counterparts within the same group to strengthen collaborative intervention measures. For high‐SDI countries exhibiting deviations, policy implementation efficacy could be optimized by referencing frontier nations such as the Netherlands. Meanwhile, low‐SDI countries may adopt low‐cost intervention strategies from leading performers in their category. This approach transforms traditional descriptive comparisons of disease burdens into a prescriptive policy guidance tool, thereby offering more targeted evidential support for context‐specific interventions.

### 4.6. Predictive Analysis

The projected decline in overall and female mortality rates from tobacco‐attributable T2DM reflects the ongoing benefits of tobacco control measures and advancements in diabetes care, though the stable male mortality rate suggests lingering impacts of historical tobacco exposure among men. In contrast, the projected rise in DALYs rates—even as mortality decreases—highlights that while fewer people may die from tobacco‐related T2DM, the disease will increasingly cause long‐term disability and health loss, likely driven by factors such as population aging, prolonged disease duration, and the cumulative effects of tobacco exposure on T2DM complications. This divergence between mortality and DALYs trends underscores the need to prioritize not only reducing T2DM‐related deaths but also mitigating morbidity, with targeted efforts for men who face more persistent mortality risks and broader strategies to address the growing burden of disability across genders.

Model projections represents a critical intervention window to prevent a resurgence in disease burden: Policymakers can leverage this projection to proactively advance tobacco tax reforms before burden deterioration occurs (e.g., drawing on experiences from high‐SDI regions where tiered tax increases have reduced tobacco accessibility) and expand coverage of smoking cessation services among middle‐aged and elderly T2DM patients (e.g., integrating primary diabetes management with smoking cessation hotline services). Special emphasis should be placed on strengthening interventions targeting male populations, as projections show stable mortality rates alongside persistently high baseline figures, thereby avoiding the reversal of stable trends into an upward trajectory.

Furthermore, mitigating the adverse impacts of tobacco exposure on T2DM may further yield savings in potential healthcare expenditures. Tobacco synergizes with multiple factors to exacerbate healthcare expenditures associated with T2DM. A study conducted in Southwest China revealed that smoking history, the number of comorbidities (e.g., hypertension and nephropathy), and the Charlson Comorbidity Index collectively contribute to increased hospitalization costs, with diagnostic‐related costs accounting for the highest proportion (≥ 25%) [70]. The additive effects of tobacco exposure and metabolic syndrome (e.g., obesity and insulin resistance) further intensify inflammatory processes and escalate the utilization of healthcare resources [71]. Additionally, research in Gulf Cooperation Council countries demonstrated that the total economic burden of smoking and secondhand smoke constitutes 1.04% of GDP [72]. While lifestyle interventions (including smoking cessation) incur moderate costs (annual per capita expenditure ranging from $131 to $461), they yield modest improvements in glycemic control, thereby validating their potential for long‐term cost savings [73].

These findings underscore the importance of continuous monitoring and adaptation of public health policies. While the downward trend in mortality rates is encouraging, they also highlight the need for sustained efforts to further alleviate the burden of tobacco exposure–related T2DM. Strengthening tobacco cessation programs, enhancing diabetes management, and addressing gender‐specific risk factors would contribute to more significant reductions in both mortality rates and DALYs. Furthermore, further research into the specific factors influencing DALYs in males could improve the accuracy of future predictions and inform more tailored interventions. However, the widening of prediction intervals indicates increased uncertainty in the future, necessitating ongoing monitoring to address potential risk factors.

### 4.7. Association With the Epidemiology of T2DM

Findings from previous epidemiological studies on middle‐aged and elderly individuals aged ≥ 55 years with T2DM have revealed that the absolute numbers and rates of deaths and DALYs attributed to T2DM in this population exhibit certain similarities to those caused by tobacco exposure–related T2DM, in terms of temporal trends, gender differences, and geographical distributions. From the perspective of epidemiological transition, the paradoxical phenomenon—an increase in the absolute number of global tobacco exposure–related T2DM deaths accompanied by a decline in rate—essentially reflects the superposition of demographic transition and the effects of disease interventions [74]. According to the epidemiological transition theory proposed by Omran, most regions worldwide have now entered the “chronic disease–dominated stage,” where population aging has emerged as the core driver of the rising absolute burden of chronic diseases [75]. Notably, the middle‐aged and elderly population itself constitutes a high‐risk group for both T2DM and long‐term tobacco exposure, directly contributing to the increase in the absolute number of deaths. Concurrently, the decline in rate underscores the effectiveness of primary prevention and healthcare interventions.

### 4.8. Limitations

The study has the following limitations: First, national‐level aggregated data obscures urban–rural disparities. Additionally, the SDI fails to capture nuanced factors such as cultural norms or the impacts of climate change. Second, the APC model faces multicollinearity challenges. Although the intrinsic estimator method was applied, residual bias may still persist when interpreting long‐term cohort trends. Third, in the frontier analysis, the assumption that frontier countries represent optimal management does not account for genetic susceptibility (e.g., in Pacific island nations) or the influence of colonial history on healthcare systems. Thus, localized validation of policy recommendations is required. Fourth, projections from the BAPC model are based on the assumption that current tobacco control intensity and healthcare standards remain unchanged. The model does not consider the potential impacts of future factors on disease burden, such as the relaxation of tobacco control policies, the popularization of emerging tobacco products, or public health emergencies [76]. Additionally, in the population‐level GBD data, residual confounding may exist due to unmeasured individual factors (e.g., smoking intensity), and causal relationships may not be established. As an ecological analysis, it carries the risk of ecological fallacy when inferring associations at the individual level.

## 5. Conclusion

Based on data from the GBD 2021 study, this research conducted a systematic analysis of the global burden of tobacco exposure–induced T2DM among middle‐aged and older adults from 1990 to 2021. Notable regional disparities were identified, with high–middle‐SDI regions shouldering the greatest burden in terms of mortality rate and DALYs. Males exhibited a significantly higher burden than females, whereas earlier birth cohorts (i.e., elderly individuals) were confronted with considerable risks—underscoring the necessity of enhanced individualized management. Frontier analysis demonstrated differences in disease burden across nations, stressing the requirement for targeted interventions. Forecasts extending to 2042 suggested that the mortality rate would sustain a downward trend, while the DALYs rate would keep rising steadily. These findings highlight the pivotal role of socioeconomic factors and customized interventions (e.g., reinforcing tobacco control policies and improving healthcare services) in mitigating the global burden of tobacco exposure–related T2DM.

## Author Contributions

Yujun He: writing—review and editing, writing—original draft, conceptualization, methodology, formal analysis, supervision. Minhui Liu: writing—review and editing, conceptualization, visualization. Bowen Xing: writing—review and editing, data curation. Hui Xu: writing—review and editing, investigation, validation. Jiujie He: writing—review and editing, formal analysis. Wei Mai: writing—review and editing, visualization. Simin Qin: writing—review and editing, investigation. Zhenyi Luo: writing—review and editing, formal analysis, data curation. Xiaoyi Wang: writing—review and editing, methodology, data curation. Yi Xu: writing—review and editing, project administration, funding acquisition. Yuping Ye: writing—review and editing, data curation, validation, investigation, visualization.

## Funding

The study is supported by the third round of Taizhou Traditional Chinese Medicine (Integrated Traditional Chinese and Western Medicine) key (supported) disciplines, Tai Wei Fa [2020] 52; the 2026 Annual General Program of Zhejiang Provincial Traditional Chinese Medicine Science and Technology Project, 2026ZL1028; the Joint Project of Scientific and Technological Innovation of the Health Commission of Hainan Province, No. WSJK2024QN123; the General Project of Scientific Research Program of Hunan Provincial Administration of Traditional Chinese Medicine, B2024153; the Youth Program of Scientific Research Foundation of Guangxi Medical University Cancer Hospital, 2023‐9; the Youth Fund of Guangxi Medical University, GXMUYSF202445; the Collaborative Research Fund for Young Scientists Supported by Chongqing Bishan District Bureau of Science and Technology and Chongqing University of Chinese Medicine, BSLHZX028; and the Excellent Doctoral Research Startup Fund, 2023‐1.

## Ethics Statement

The authors have nothing to report.

## Consent

The authors have nothing to report.

## Conflicts of Interest

The authors declare no conflicts of interest.

## Supporting information


**Supporting Information** Additional supporting information can be found online in the Supporting Information section. (Supporting Information) Table S1: The case number and per 100,000 aged ≥ 55 population of deaths of T2DM attributable to smoking in 1990 and 2021, and its temporal trends from 1990 to 2021, categorized by global, SDI, and 21 regions. Table S2: The case number and per 100,000 aged ≥ 55 population of DALYs of T2DM attributable to smoking in 1990 and 2021, and its temporal trends from 1990 to 2021, categorized by global, SDI, and 21 regions. Table S3: Findings on death rates per 100,000 aged ≥ 55 population in frontier analysis. Table S4: Findings on DALYs rates per 100,000 aged ≥ 55 population in frontier analysis. Figure S1: The trends of number in deaths for T2DM attributable to tobacco exposure in middle‐aged and elderly patients categorized by global and five SDI regions from 1990 to 2021. Figure S2: The trends of rate per 100,000 aged ≥ 55 population in deaths for T2DM attributable to tobacco exposure in middle‐aged and elderly patients categorized by global and five SDI regions from 1990 to 2021. Figure S3: The trends of rate per 100,000 aged ≥ 55 population in DALYs for T2DM attributable to tobacco exposure in middle‐aged and elderly patients categorized by global and five SDI regions from 1990 to 2021. Figure S4: Results of age–period–cohort analysis for DALYs: trends and deviations in rates per 100,000 aged ≥ 55 population across age groups (A), birth cohort (B), and year (C) dimensions. Figure S5: Relationship between SDI and the burden of T2DM attributable to tobacco exposure in middle‐aged and elderly patients: results of deaths (a) and DALYs (b) across 204 countries and territories. The shaded area represents the corresponding 95% UI. Figure S6: Frontier analysis, represented by the solid black lines, explores the relationship between SDI and rate per 100,000 aged ≥ 55 population for DALYs in the context of T2DM attributable to tobacco exposure in middle‐aged and elderly patients. The color gradient in Graph A illustrates the progression of years, ranging from light shades representing 1990 to the darkest shades denoting 2021. In Graph B, each dot signifies a specific country or territory for the year 2021, with the top 15 countries displaying the most significant deviation from the frontier labeled in black. Countries with low SDI (> 0.455) and minimal deviation from the frontier are highlighted in blue, while those with high SDI (> 0.805) and notable deviation for their developmental level are emphasized in red. The direction of change from 1990 to 2021 in rate per 100,000 aged ≥ 55 population is indicated by the color of the dots: Red dots represent decrease, while blue dots signify increase. Figure S7: Predicted trends of T2DM attributable to tobacco exposure over the next 15 years (2022–2042) (A, rate per 100,000 aged ≥ 55 population of DALYs of both; B, rate per 100,000 aged ≥ 55 population of DALYs of male; C, rate per 100,000 aged ≥ 55 population of DALYs of female). Dark blue lines represent the true trend during 1990–2021; black lines and dark blue shaded regions represent the predicted trend and its 95% CI.

## Data Availability

The datasets utilized in this investigation are accessible in open repositories. The repository names and accession numbers are provided below: All data may be accessed via the IHME website (https://vizhub.healthdata.org/gbd-results/).
