# Supplementary material for: Global Mortality and Disability‐Adjusted Life Years Attributable to Tobacco Exposure Among Middle‐Aged and Older Adults With Type 2 Diabetes, 1990–2021: A Systematic Analysis of GBD 2021 Data With Projections to 2042
Source: J Diabetes Res. 2026 Mar 12;2026:5522115. doi: 10.1155/jdr/5522115 (PMC13140178; doi:10.1155/jdr/5522115)
Supplement: Supplementary file 1 — Supporting Information Additional supporting information can be found online in the Supporting Information section. (Supporting Information) Table S1: The case number and per 100,000 aged ≥ 55 population of deaths of T2DM attributable to smoking in 1990 and 2021, and its temporal trends from 1990 to 2021, categorized by global, SDI, and 21 regions. Table S2: The case number and per 100,000 aged ≥ 55 population of DALYs of T2DM attributable to smoking in 1990 and 2021, and its temporal trends from 1990 to 2021, categorized by global, SDI, and 21 regions. Table S3: Findings on death rates per 100,000 aged ≥ 55 population in frontier analysis. Table S4: Findings on DALYs rates per 100,000 aged ≥ 55 population in frontier analysis. Figure S1: The trends of number in deaths for T2DM attributable to tobacco exposure in middle‐aged and elderly patients categorized by global and five SDI regions from 1990 to 2021. Figure S2: The trends of rate per 100,000 aged ≥ 55 population in deaths for T2DM attributable to tobacco exposure in middle‐aged and elderly patients categorized by global and five SDI regions from 1990 to 2021. Figure S3: The trends of rate per 100,000 aged ≥ 55 population in DALYs for T2DM attributable to tobacco exposure in middle‐aged and elderly patients categorized by global and five SDI regions from 1990 to 2021. Figure S4: Results of age–period–cohort analysis for DALYs: trends and deviations in rates per 100,000 aged ≥ 55 population across age groups (A), birth cohort (B), and year (C) dimensions. Figure S5: Relationship between SDI and the burden of T2DM attributable to tobacco exposure in middle‐aged and elderly patients: results of deaths (a) and DALYs (b) across 204 countries and territories. The shaded area represents the corresponding 95% UI. Figure S6: Frontier analysis, represented by the solid black lines, explores the relationship between SDI and rate per 100,000 aged ≥ 55 population for DALYs in the context of T2DM attributable to tobacco expo [file JDR-2026-5522115-s001.pdf]

Table S1 The case number and rate per 100,000 aged ≥55 population of deaths of T2DM attributable to tobacco exposure in 1990 and 2021, and its temporal trends from 1990 to 2021, categorized by global, SDI and 21 regions.

Location name	1990		2021		1990-2021		
	**Case number (95% UI)**	**rate per 100,000 aged ≥55 population (95% UI)**	**Case number (95% UI)**	**rate per 100,000 aged ≥55 population (95% UI)**	**EAPC (95% CI)**	**RC of numbers (%)**	**RC of rate per 100,000 aged ≥55 population (%)**
Global	69998.24(45199.87-92972.92)	10.43(6.73-13.85)	140767.54(88450.01-194600.29)	9.47(5.95-13.10)	-0.48(-0.58, -0.39)	101.10	-9.13
**Sex**							
Male	40228.66(29142.24-51114.97)	12.92(9.36-16.41)	88542.99(62436.21-115545.20)	12.66(8.93-16.52)	-0.20(-0.27, -0.12)	120.10	-1.99
Female	29769.59(15693.89-43208.86)	8.27(4.36-12.00)	52224.55(25799.19-79543.33)	6.64(3.28-10.11)	-0.95(-1.07, -0.82)	75.43	-19.71
**SDI**							
High SDI	15394.46(10787.75-20237.58)	8.26(5.79-10.85)	16455.00(11087.95-22530.61)	4.77(3.21-6.53)	-2.27(-2.51, -2.03)	6.89	-42.23
High-middle SDI	13944.63(8912.71-1	8.08(5.17-10.88)	25441.96(15885.86-34	7.34(4.58-10.02)	-0.41(-0.53,	82.45	-9.2

	8764.88)		743.33)		-0.28)		
Middle SDI	21660.69(13631.32-29094.29)	12.48(7.85-16.76)	51506.67(31672.94-71249.51)	10.96(6.74-15.16)	-0.61(-0.71,-0.50)	137.79	-12.16
Low-middle SDI	14614.50(9174.07-19770.92)	14.50(9.10-19.61)	38711.12(23394.35-54063.06)	16.06(9.70-22.43)	0.34(0.30,0.38)	164.88	10.75
Low SDI	4277.58(2648.23-6024.64)	11.47(7.10-16.15)	8487.56(4978.16-12224.00)	10.34(6.07-14.90)	-0.42(-0.49,-0.35)	98.42	-9.79
**Regions**							
Andean Latin America	229.40(140.81-321.93)	6.84(4.20-9.59)	615.44(383.55-892.25)	6.21(3.87-9.01)	-0.55(-0.69,-0.40)	168.29	-9.12
Australasia	238.29(163.65-325.52)	6.05(4.15-8.26)	260.71(160.21-374.87)	2.95(1.81-4.24)	-2.93(-3.26,-2.61)	9.41	-51.21
Caribbean	844.61(534.61-1158.36)	19.60(12.40-26.88)	1150.63(702.99-1628.14)	12.43(7.59-17.59)	-1.74(-1.87,-1.61)	36.23	-36.59
Central Asia	422.52(251.57-595.	5.28(3.15-7.44)	1271.45(748.45-1834.8	8.74(5.14-12.61)	1.41(0.81,2.	200.92	65.42

	08)		6)		01)		
Central Europe	2357.86(1557.85-3153.54)	8.89(5.87-11.89)	3228.60(2051.54-4477.18)	8.72(5.54-12.09)	0.04(-0.11,0.19)	36.93	-1.93
Central Latin America	3538.81(2228.87-4818.48)	26.08(16.42-35.51)	6112.38(3812.64-8429.43)	14.29(8.92-19.71)	-2.37(-2.61,-2.12)	72.72	-45.19
Central Sub-Saharan Africa	472.57(282.20-692.90)	12.57(7.50-18.43)	908.27(551.36-1346.16)	10.07(6.11-14.92)	-0.88(-1.06,-0.70)	92.2	-19.91
East Asia	11192.36(7226.02-15088.30)	7.51(4.85-10.13)	26948.94(16554.23-37758.49)	6.87(4.22-9.63)	-0.38(-0.61,-0.16)	140.78	-8.54
Eastern Europe	1022.16(615.59-1423.00)	2.09(1.26-2.91)	3381.03(2125.32-4640.96)	5.45(3.42-7.48)	1.91(0.73,3.12)	230.77	160.51
Eastern Sub-Saharan Africa	1650.31(1056.85-2257.12)	13.57(8.69-18.55)	2557.57(1610.17-3605.36)	9.46(5.96-13.33)	-1.44(-1.54,-1.34)	54.98	-30.27
High-income Asia Pacific	2286.97(1557.16-3014.65)	6.54(4.45-8.62)	1810.11(1197.68-2520.99)	2.57(1.70-3.58)	-3.38(-3.57,-3.18)	-20.85	-60.74
High-i	5463.0	9.43(6.75-1	6146.00	5.46(3.69-7.	-2.6	12.5	-42.09

ncome North America	4(3909.71-7202.04)	2.43)	(4155.07-8444.30)	50)	9(-3.17,-2.21)	0	
North Africa and Middle East	3998.62(2395.33-5656.30)	14.15(8.47-20.01)	11669.04(6903.33-16673.84)	15.31(9.06-21.87)	0.49(0.37,0.60)	191.83	8.2
Oceania	277.25(169.39-398.46)	57.63(35.21-82.82)	710.86(400.39-1025.87)	57.60(32.44-83.12)	-0.14(-0.25,-0.03)	156.4	-0.05
South Asia	12538.32(7748.89-17241.80)	13.21(8.16-18.16)	35526.51(21111.59-51041.57)	14.31(8.50-20.56)	0.17(0.09,0.24)	183.34	8.34
Southeast Asia	8146.51(5132.40-10959.39)	19.24(12.12-25.88)	20534.36(12672.88-28533.05)	17.93(11.06-24.91)	-0.40(-0.52,-0.29)	152.06	-6.83
Southern Latin America	1047.88(657.82-1452.95)	13.23(8.30-18.34)	1149.58(698.42-1618.46)	7.81(4.75-11.00)	-1.96(-2.27,-1.65)	9.70	-40.95
Southern Sub-Saharan Africa	1239.34(775.76-1746.18)	28.01(17.53-39.46)	2896.06(1683.28-4097.74)	29.75(17.29-42.09)	0.34(-0.08,0.75)	133.68	6.21
Tropical Latin Ameri	3499.12(2236.48-4673.82)	23.11(14.77-30.87)	5561.39(3490.87-7763.83)	12.55(7.88-17.53)	-2.18(-2.29,-2.0	58.94	-45.67

ca					7)		
Western Europe	8563.87(5822.70-11434.94)	8.82(6.00-11.77)	6190.02(3978.03-8592.94)	4.15(2.67-5.76)	-2.49(-2.62, -2.36)	-27.72	-52.93
Western Sub-Saharan Africa	968.43(551.99-1441.86)	6.71(3.82-9.99)	2138.57(1168.70-3090.63)	6.65(3.64-9.62)	-0.10(-0.33, 0.13)	120.83	-0.83

Abbreviations: RC, relative change; EAPC, estimated annual percentage change; SDI, sociodemographic index; UI, uncertainty interval; CI, confidence interval.

Table S2 The case number and rate per 100,000 aged ≥55 population of DALYs of T2DM attributable to tobacco exposure in 1990 and 2021, and its temporal trends from 1990 to 2021, categorized by global, SDI and 21 regions.

Location name	1990		2021		1990-2021		
	Case number (95% UI)	rate per 100,000 aged ≥55 population (95% UI)	Case number (95% UI)	rate per 100,000 aged ≥55 population (95% UI)	EAPC (95% CI)	RC of numbers (%)	RC of rate per 100,000 aged ≥55 population (%)
Global	2468668.57(1623485.26-3358211.49)	367.68(241.80-500.16)	5934138.22(3798274.69-8469349.66)	399.34(255.61-569.95)	0.08(0.01, 0.15)	140.38	8.61
**Sex**							
Male	1484366.43(1091194.64-1916973.55)	476.55(350.32-615.43)	3844532.06(2703707.36-5193668.83)	549.60(386.51-742.46)	0.31(0.24, 0.37)	159.00	15.33
Female	984302.14(526147.93-1456997.75)	273.46(146.18-404.79)	2089606.17(1059413.74-3222863.26)	265.69(134.71-409.79)	-0.32(-0.41, -0.24)	112.29	-2.84
**SDI**							
High SDI	569047.29(401153.45-758723.40)	305.18(215.14-406.90)	1028673.91(686991.42-1464521.32)	298.16(199.12-424.48)	-0.38(-0.51, -0.25)	80.77	-2.30
High-middle	545521.14(3515	316.20(203.75-436.01)	1236647.32(7783	356.71(224.52-512.28)	0.24(0.	126.69	12.81

SDI	23.01-752228.24)		77.05-1775955.65)		16, 0.33)		
Middle SDI	772354.44(490073.35-1071891.67)	445.01(282.36-617.59)	2063377.06(1282352.55-2930464.32)	439.15(272.92-623.69)	-0.28(-0.36, -0.19)	167.15	-1.32
Low-middle SDI	450990.05(286748.70-611656.37)	447.41(284.47-606.80)	1315203.92(810747.49-1849719.19)	545.54(336.29-767.25)	0.61(0.58, 0.65)	191.63	21.93
Low SDI	126962.66(79724.75-176770.87)	340.31(213.69-473.81)	283319.80(173614.04-403466.26)	345.27(211.57-491.68)	-0.09(-0.15, -0.03)	123.15	1.46
**Regions**							
Andean Latin America	7409.05(4626.39-10437.46)	220.78(137.86-311.02)	23807.49(14906.43-34439.05)	240.32(150.47-347.64)	-0.00(-0.11, 0.11)	221.33	8.85
Australasia	8467.69(5820.38-11746.68)	214.94(147.74-298.18)	13486.54(8631.38-19647.28)	152.66(97.70-222.40)	-1.45(-1.59, -1.32)	59.27	-28.98
Caribbean	26764.45(17496.76-37084.24)	621.02(405.98-860.48)	49518.91(31248.50-70757.60)	534.85(337.51-764.25)	-0.74(-0.84, -0.65)	85.02	-13.88
Centra	17290.8	216.19(129	59364.0	408.01(241	1.9	243.	88.73

l Asia	7(10349.19-24575.71)	.40-307.27)	9(35109.10-87016.87)	.30-598.06)	0(1.52, 2.28)	33	
Central Europe	105497.18(69761.96-145280.30)	397.80(263.05-547.81)	159350.43(104136.78-225681.86)	430.35(281.24-609.49)	0.34(0.27, 0.41)	51.05	8.18
Central Latin America	111557.75(71145.36-153117.45)	822.08(524.28-1128.34)	209854.42(130235.81-294454.57)	490.70(304.53-688.52)	-2.12(-2.33, -1.90)	88.11	-40.31
Central Sub-Saharan Africa	13875.67(8356.54-20186.68)	369.00(222.23-536.84)	30999.96(19043.38-44382.90)	343.55(211.04-491.86)	-0.34(-0.55, -0.13)	123.41	-6.90
East Asia	501418.02(320701.71-700384.39)	336.63(215.30-470.20)	1362861.40(850727.35-1989297.09)	347.56(216.96-507.32)	-0.09(-0.19, 0.01)	171.80	3.25
Eastern Europe	62773.28(37118.58-92375.24)	128.39(75.92-188.93)	161135.99(104280.63-229312.55)	259.57(167.98-369.39)	1.78(1.45, 2.11)	156.70	102.17
Eastern Sub-Saharan Africa	43820.64(27972.19-60022.84)	360.20(229.93-493.38)	74199.08(47038.27-104132.18)	274.43(173.97-385.14)	-1.15(-1.25, -1.05)	69.32	-23.81
High-income	114675.94(7779	327.94(222.46-455.28)	179039.49(1124	253.94(159.53-385.87)	-1.27(-1	56.13	-22.56

Asia Pacific	1.48-159201.94)		72.00-272050.46)		.45, -1.08)		
High-income North America	201877.25(141893.04-270828.83)	348.50(244.95-467.53)	434933.75(290492.07-626437.92)	386.49(258.14-556.66)	-0.08(-0.27, 0.12)	115.44	10.9
North Africa and Middle East	132293.29(81390.31-186361.48)	468.07(287.97-659.36)	527537.28(309521.76-766192.49)	692.00(406.02-1005.06)	1.35(1.28, 1.42)	298.76	47.84
Oceania	8036.43(5020.88-11441.44)	1670.47(1043.65-2378.24)	23307.73(13744.38-33355.03)	1888.54(1113.66-2702.64)	0.28(0.20, 0.37)	190.03	13.05
South Asia	388356.34(242633.41-532035.17)	409.04(255.56-560.38)	1145747.15(682059.32-1630047.77)	461.45(274.70-656.50)	0.26(0.18, 0.33)	195.02	12.81
Southeast Asia	249973.01(159814.48-334198.26)	590.38(377.44-789.29)	742791.17(467227.59-1032867.37)	648.42(407.86-901.64)	0.12(0.05, 0.18)	197.15	9.83
Southern Latin America	33898.98(21984.15-46560.62)	427.94(277.53-587.78)	57098.46(35558.99-82406.34)	388.00(241.63-559.97)	-0.57(-0.75, -0.38)	68.44	-9.33
Southern Sub-S	33682.97(21171.37-4648	761.24(478.47-1050.50)	85736.34(51151.23-1212	880.68(525.42-1245.94)	0.62(0.31,	154.54	15.69

aharan Africa	2.07)		95.64)		0.93)		
Tropical Latin America	118058.70(76672.03-159687.02)	779.71(506.38-1054.64)	213658.29(136806.17-307597.83)	482.32(308.83-694.39)	-1.77(-1.88, -1.67)	80.98	-38.14
Western Europe	260964.53(182315.36-349127.57)	268.72(187.74-359.51)	307908.91(205350.85-442522.58)	206.46(137.70-296.73)	-0.97(-1.06, -0.89)	17.99	-23.17
Western Sub-Saharan Africa	27976.53(15933.43-40993.21)	193.80(110.38-283.98)	71801.36(40696.10-105405.40)	223.38(126.61-327.93)	0.35(0.15, 0.54)	156.65	15.26

Abbreviations: RC, relative change; EAPC, estimated annual percentage change; SDI, sociodemographic index; UI, uncertainty interval; CI, confidence interval.

Table S3 Findings on deaths rates for T2DM attributable to tobacco exposure per 100,000 aged ≥55 population in frontier analysis

location name	year	val	SDI	frontier	trend
Kiribati	2021	158.41	0.53	1.98	Increase
Fiji	2021	126.18	0.68	1.92	Increase
Nauru	2021	89.91	0.63	1.98	Increase
Tonga	2021	77.77	0.63	1.98	Increase
Micronesia (Federated States of)	2021	75.91	0.59	1.97	Increase
Solomon Islands	2021	73.99	0.43	1.98	Increase
Mauritius	2021	69.53	0.72	1.72	Increase
Marshall Islands	2021	66.00	0.57	1.99	Increase
American Samoa	2021	63.13	0.72	1.60	Increase
Cook Islands	2021	62.17	0.78	1.22	Decrease
Tuvalu	2021	62.15	0.58	1.99	Increase
Niue	2021	58.69	0.73	1.54	Increase
Samoa	2021	57.16	0.59	1.98	Increase
Lesotho	2021	55.34	0.51	1.99	Increase
Tokelau	2021	47.07	0.69	1.92	Increase
Somalia	2021	13.38	0.08	13.38	Decrease
Niger	2021	3.76	0.17	3.76	Decrease
Ethiopia	2021	4.92	0.36	1.98	Decrease
Chad	2021	7.01	0.24	3.76	Increase
Benin	2021	5.37	0.37	1.97	Decrease
Taiwan (Province of China)	2021	14.28	0.87	0.99	Decrease
Denmark	2021	7.11	0.90	1.05	Decrease
Republic of Korea	2021	6.36	0.89	1.00	Decrease
United States of America	2021	5.68	0.86	1.03	Decrease
Austria	2021	5.09	0.85	1.06	Decrease

Table S4 Findings on DALYs rates for T2DM attributable to tobacco exposure per 100,000 aged ≥55 population in frontier analysis

location name	year	val	SDI	frontier	trend
Kiribati	2021	4882.27	0.53	77.85	Increase
Fiji	2021	3728.70	0.68	77.40	Increase
Nauru	2021	2937.11	0.63	78.11	Increase
Micronesia (Federated States of)	2021	2640.66	0.59	77.86	Increase
Marshall Islands	2021	2314.02	0.57	77.96	Increase
American Samoa	2021	2291.87	0.72	78.08	Increase
Solomon Islands	2021	2253.73	0.43	77.86	Increase
Tonga	2021	2236.93	0.63	77.92	Increase
Mauritius	2021	2138.99	0.72	77.89	Increase
Cook Islands	2021	1972.46	0.78	77.67	Decrease
Samoa	2021	1958.96	0.59	77.53	Increase
Tuvalu	2021	1934.34	0.58	77.72	Increase
Niue	2021	1873.68	0.73	77.61	Increase
Bahrain	2021	1608.10	0.75	77.75	Increase
Tokelau	2021	1602.99	0.69	77.94	Increase
Somalia	2021	402.31	0.08	402.31	Decrease
Niger	2021	142.12	0.17	124.44	Increase
Ethiopia	2021	149.91	0.36	77.44	Decrease
Burundi	2021	208.95	0.29	123.59	Decrease
Madagascar	2021	179.01	0.40	77.68	Decrease
Taiwan (Province of China)	2021	505.43	0.87	77.90	Decrease
Republic of Korea	2021	412.45	0.89	77.91	Decrease
United States of America	2021	399.58	0.86	77.72	Increase
Canada	2021	279.59	0.87	77.60	Decrease
Switzerland	2021	242.25	0.93	77.56	Decrease

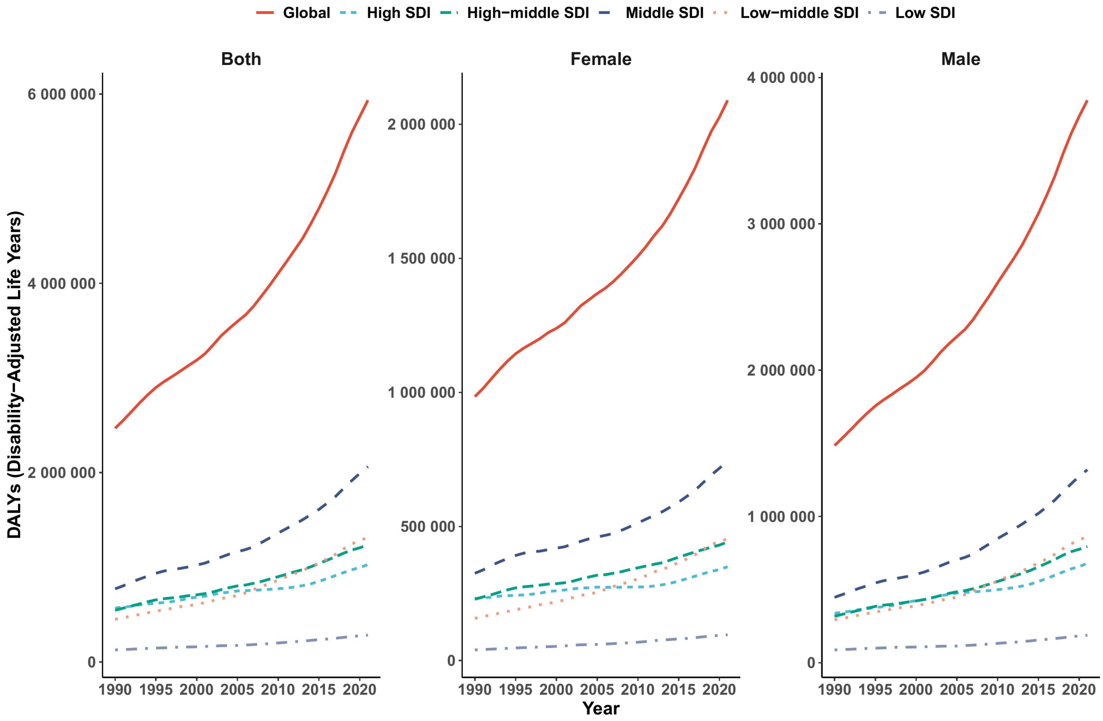

Figure S1 The trends of number in deaths for T2DM attributable to tobacco exposure in aged ≥55 patients categorized by global and 5 SDI regions from 1990 to 2021.

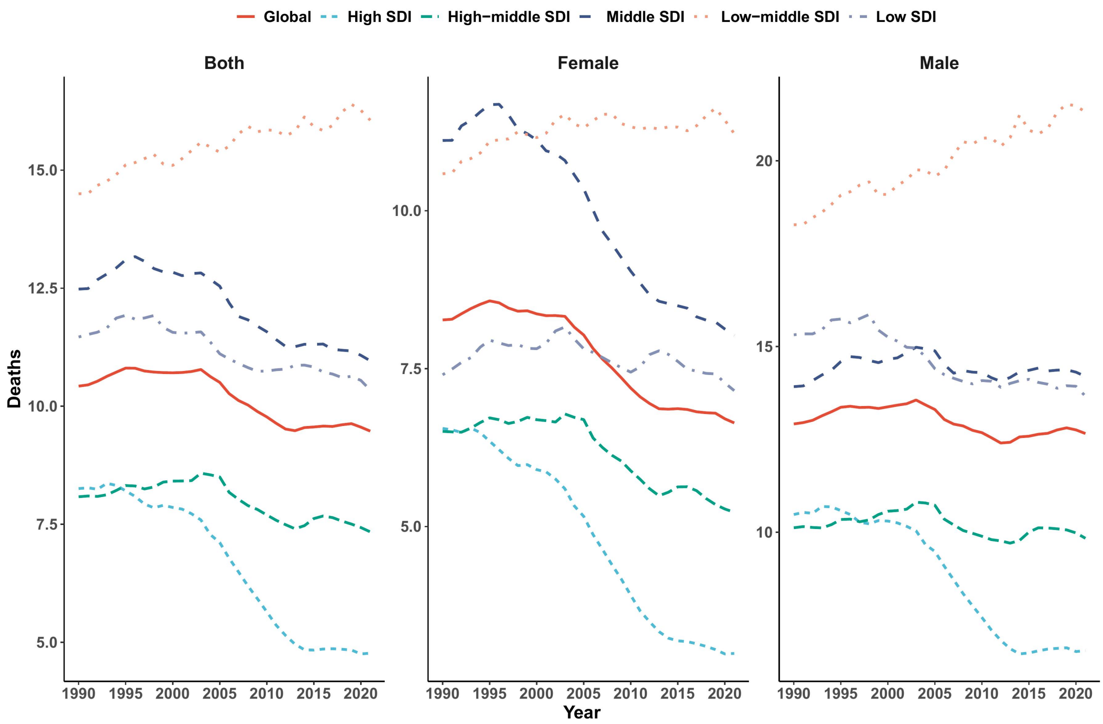

Figure S2 The trends of rate per 100,000 aged ≥55 population in deaths for T2DM attributable to tobacco exposure in middle-aged and elderly patients categorized by global and 5 SDI regions from 1990 to 2021.

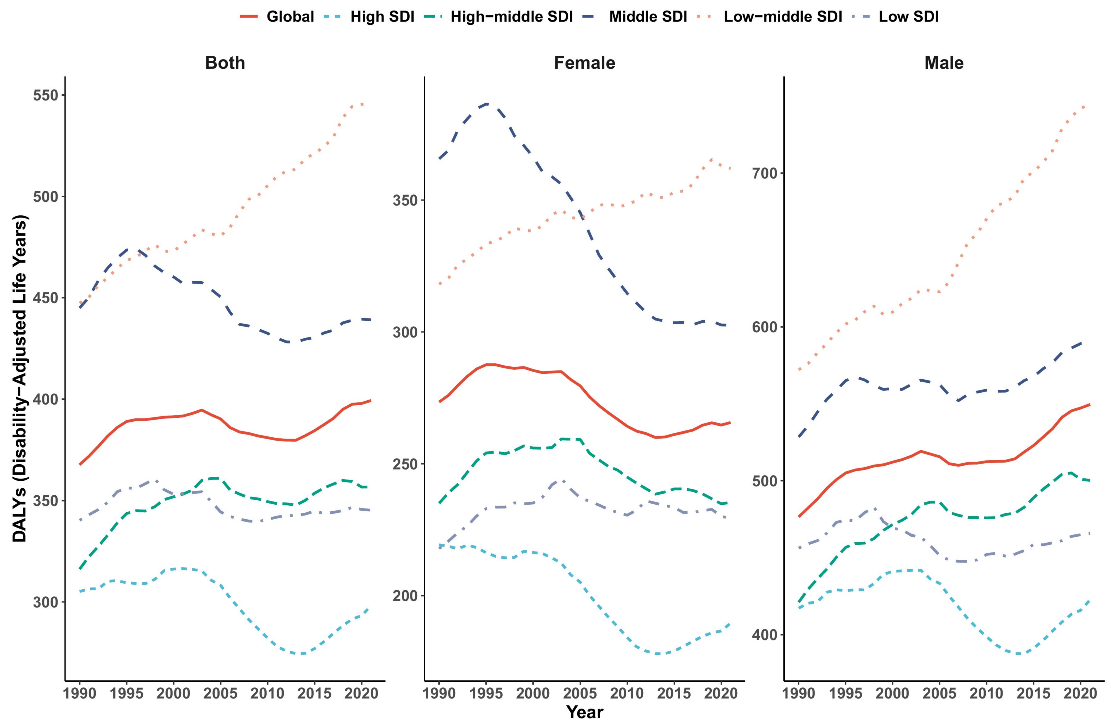

Figure S3 The trends of rate per 100,000 aged ≥55 population in DALYs for T2DM attributable to tobacco exposure in middle-aged and elderly patients categorized by global and 5 SDI regions from 1990 to 2021.

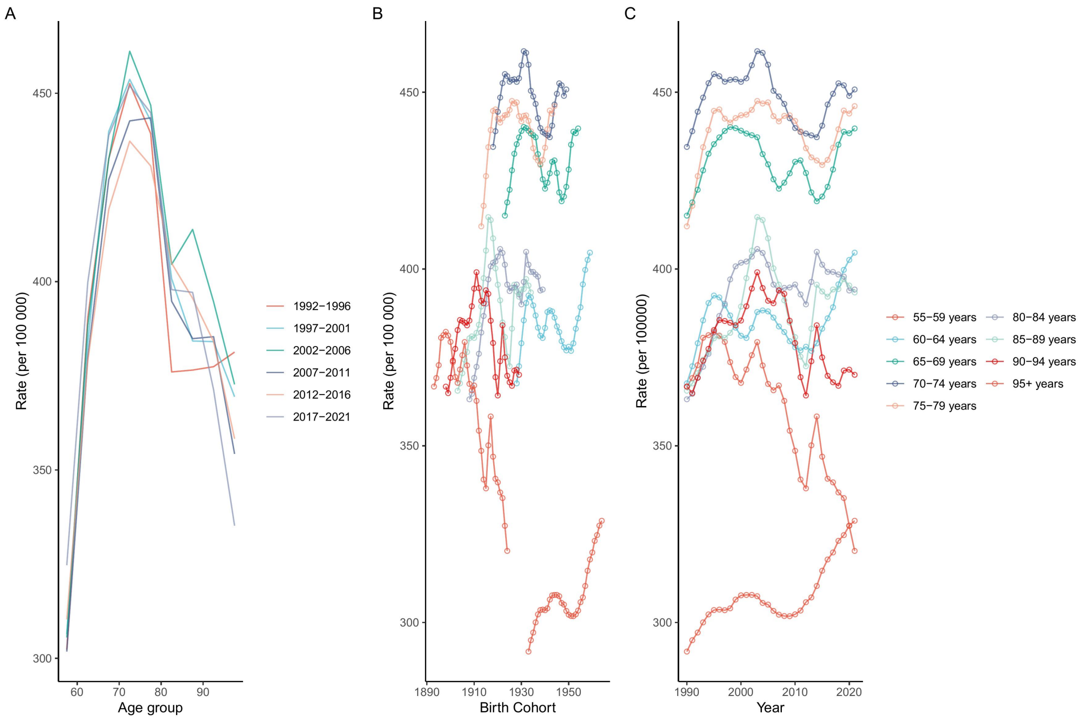

Figure S4 Results of Age-Period-Cohort Analysis for DALYs: trends and deviations in rates per 100,000 aged ≥55 population across Age groups (A), birth cohort (B), and year (C) dimensions.

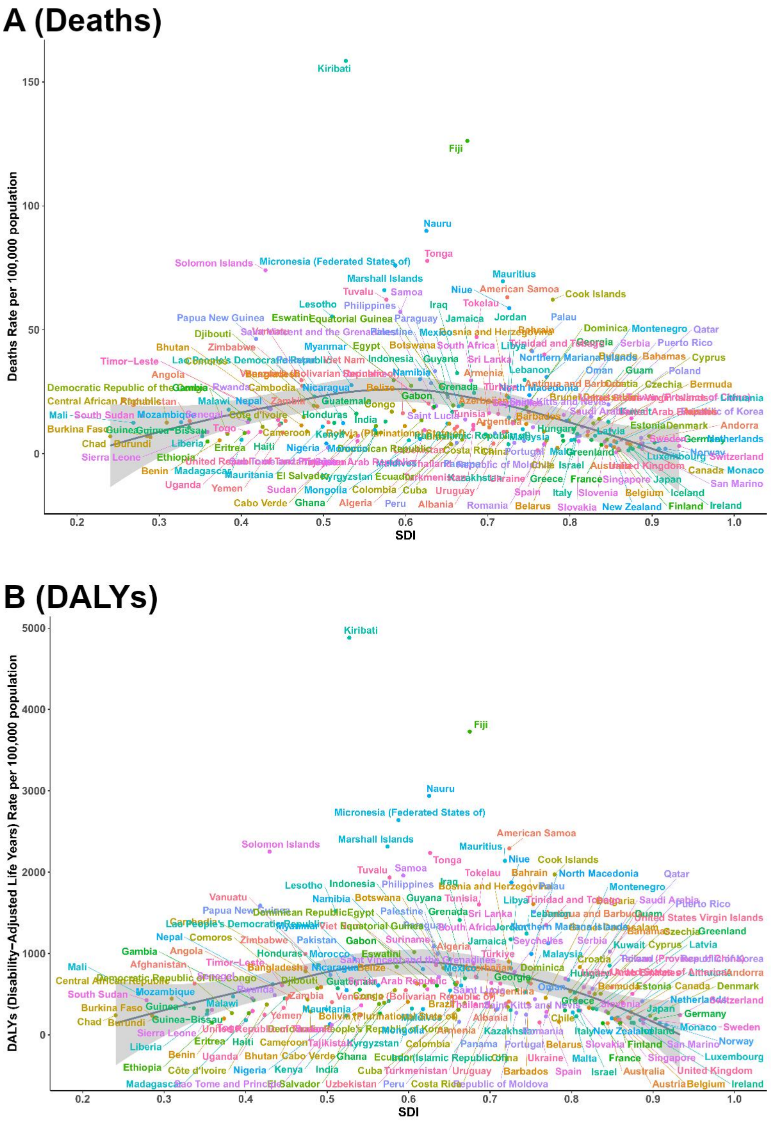

Figure S5 Relationship between SDI and the burden of T2DM attributable to tobacco exposure in middle-aged and elderly patients: results of deaths (a) and DALYs (b) across 204 countries and territories. The shaded area represents the corresponding 95% CI.

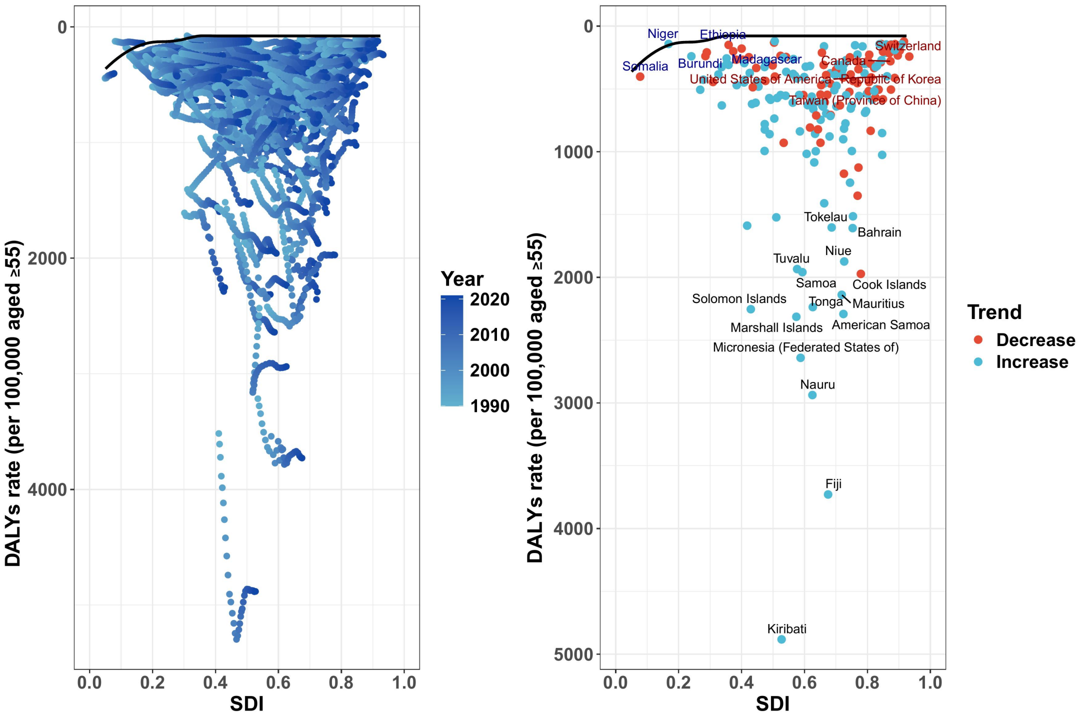

Figure S6 Frontier analysis, represented by the solid black lines, explores the relationship between SDI and rate per 100,000 aged ≥55 population per 100,000 population for DALYs in the context of T2DM attributable to tobacco exposure in middle-aged and elderly patients. The color gradient in graphs A illustrates the progression of years, ranging from light shades representing 1990 to the darkest shades denoting 2021. In graphs B, each dot signifies a specific country or territory for the year 2021, with the top 15 countries displaying the most significant deviation from the frontier labeled in black. Countries with low SDI (> 0.455) and minimal deviation from the frontier are highlighted in blue, while those with high SDI (> 0.805) and notable deviation for their developmental level are emphasized in red. The direction of change from 1990 to 2021 in rate per 100,000 aged ≥55 population per 100,000 population is indicated by the color of the dots: red dots represent decrease, while blue dots signify increase.

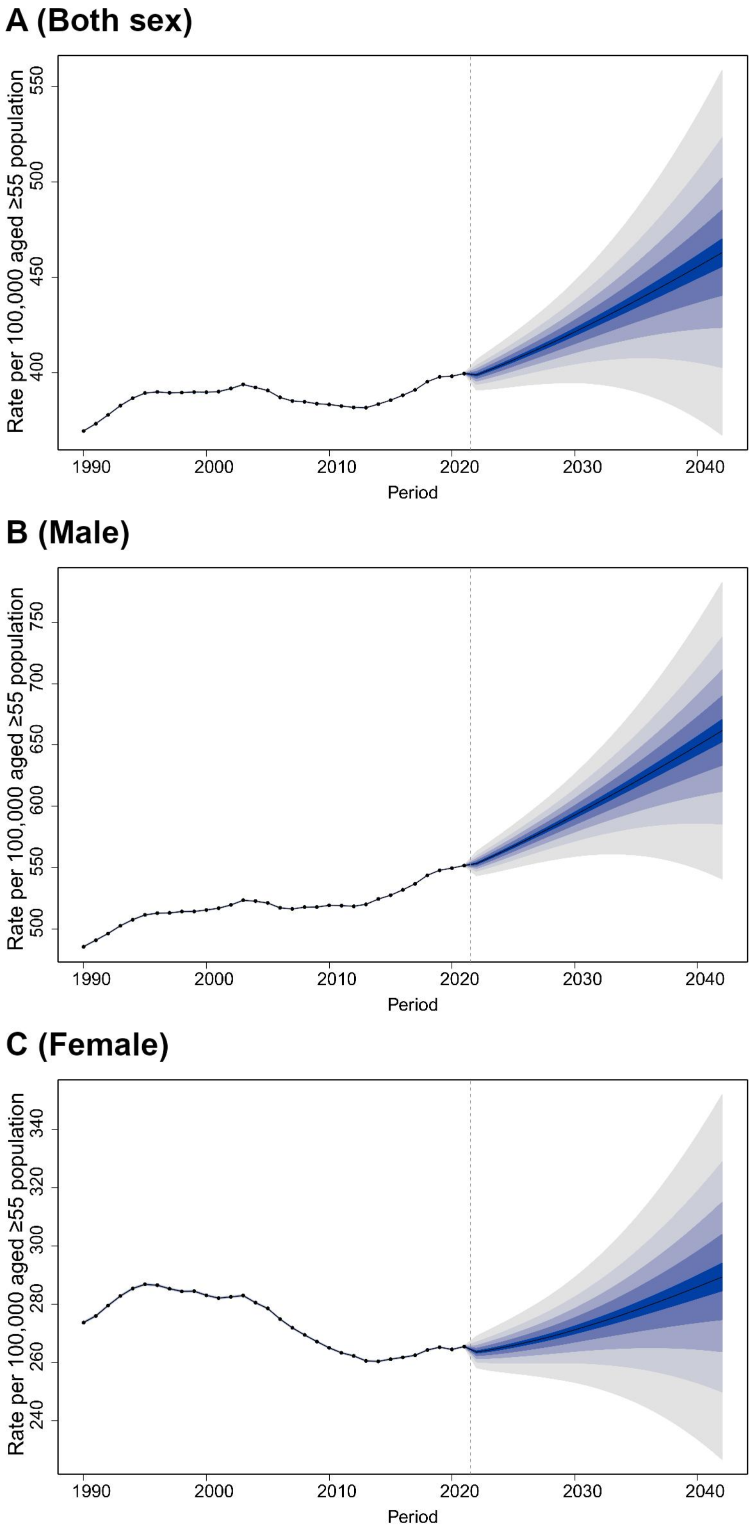

Figure S7 Predicted trends of T2DM attributable to tobacco exposure over the next 15 years (2022-2042) (A. rate per 100,000 aged ≥55 population per 100,000 population of DALYs of both; B. rate per 100,000 aged ≥55 population per 100,000 population of DALYs of male; C. rate per 100,000 aged ≥55 population per 100,000 population of DALYs of female). Dark blue lines represent the true trend during

1990-2021; black lines and dark blue shaded regions represent the predicted trend and its 95% CI.

Supplementary Notes on Frontier Analytical and Computational Methods

This study employed a nonparametric approach combined with bootstrap sampling for advanced frontier analysis. The core steps are as follows (taking the calculation of Deaths as an example):

Data Screening and Merging

Samples of the mortality rate (Rate) indicator were screened from the GBD database, including populations aged 55 years and older and all genders. Data on the Socio-Demographic Index (SDI) for 2021 were merged, with the final unit of analysis being the mortality rate (val) and corresponding SDI value at the "region-year" level.

Bootstrap Sampling and Frontier Identification

A total of 100 iterations of bootstrap sampling with replacement were performed, with the sample size consistent with the original data to enhance result robustness.

For each bootstrap sample, data were sorted by SDI in ascending order and mortality rate (val) in descending order. Since a lower mortality rate indicates better health performance, "best practice points" were identified after sorting.

Identification of "super-efficient points": For each point, the cumulative minimum mortality rate (i.e., frontier value) of the preceding samples was calculated after excluding the point. If the mortality rate of the point was lower than the preceding frontier value, it was classified as a super-efficient point (to be excluded to avoid frontier distortion by extreme values).

After excluding super-efficient points, the cumulative minimum mortality rate of the remaining samples was recalculated as the frontier value for that bootstrap sample.

Frontier Value Aggregation and Visualization

Results from 100 bootstrap iterations were aggregated, and the mean frontier value was calculated by "region-year" groups as the final frontier estimate.

For visualization, LOESS (Locally Weighted Scatterplot Smoothing) was used to smooth the frontier values (span=0.2), aiming to visually present the relationship between SDI and the best-practice mortality rate.

Frontier Estimation Method and Software

This study adopted a nonparametric frontier analysis method combined with bootstrap sampling to enhance robustness. Its core logic is similar to the Free Disposal Hull (FDH) model (a type of nonparametric method that does not require presetting a

functional form): frontier was defined by the cumulative minimum mortality rate (i.e., "best practice") through sample sorting, identification, and exclusion of super-efficient points. Specific steps included: sorting by SDI, determining the frontier via iterative calculation of cumulative minimum values, and excluding extreme super-efficient points to avoid bias.

Smoothing Selection

LOESS (Locally Weighted Scatterplot Smoothing) was used for frontier value smoothing during visualization (method='loess', span=0.2) to clarify trends. A span of 0.2 means that 20% of local samples were used for weighted fitting, balancing smoothness and local trend capture.

Analysis Software

Analyses were performed using R (version 4.4.2 or higher). The data.table package was used for data processing, the parallel package for parallel bootstrap sampling, and ggplot2 for graphing.

Indicator Units

In the analysis, "val" refers to the mortality rate of populations aged 55 years and older (Deaths per 100,000 population), i.e., the number of deaths per 100,000 individuals aged 55 years and older (consistent with the definition of the "Rate" indicator in the GBD database). The frontier value shares the same unit as the mortality rate, representing the best-practice mortality rate (minimum mortality risk) at a specific SDI level.

Temporal Dimension of Frontier Calculation

Frontiers were calculated independently by year rather than merged across years. In the code, the mean frontier value was computed by grouping with by = .(location_name, year, val, SDI), ensuring that the frontier for each year was based solely on the sample characteristics of that year and reflecting the relationship between SDI and best-practice mortality rate within each year.

Sensitivity Analysis

To verify result robustness, two sensitivity analyses with alternative frontier definitions were supplemented:

Alternative Method 1 (without excluding super-efficient points): The cumulative minimum mortality rate of sorted samples was directly used as the frontier (i.e., all points were retained without excluding super-efficient points). Results showed that

the frontier trend was consistent with the original method (difference < 5%).

Alternative Method 2 (quantile regression frontier): Nonparametric quantile regression (q=0.05, i.e., 5th percentile) was used to estimate the frontier (representing that 95% of samples were above this level). The coincidence degree between the frontier curve and that of the original method reached 0.92, verifying trend stability.

Algorithm Explanation for "frontier" in Table S3

The "frontier" values in Table S3 are the means obtained via the aforementioned bootstrap nonparametric method. For each "region-year", the arithmetic mean of frontier values calculated in each of the 100 bootstrap iterations was taken as the final estimate, reflecting the best-practice mortality rate (i.e., theoretical minimum mortality risk) of the region at the corresponding year and SDI level.

Supplementary Notes on BAPC Analytical and Computational Methods

The BAPC model in this study adopts a "Poisson likelihood function", expressed as $Y_{a,p} \sim \text{Poisson}(\lambda_{a,p})$ (where $Y_{a,p}$ denotes the number of cases in age group a and period p, and $\lambda_{a,p}$ represents the incidence mean). It is paired with a "log link function" to construct a linear predictor, while incorporating a "population size offset ($\log(pop_{a,p})$)" to adjust for differences in population size across age-period groups. The specific form of the linear predictor is $\log(\lambda_{a,p}) = \log(pop_{a,p}) + \mu + \alpha_a + \beta_p + \gamma\_c$ (where μ is the global intercept, α_a the age effect, β_p the period effect, γ_c the cohort effect, and c is derived from period and age). For the prediction phase, an independent random effect $\delta_{a,p+t} \sim N(0, k\_\delta^{-1})$ is further added to adjust for overdispersion, expanding the formula to $\log(\lambda_{a,p+t}) = \log(pop_{a,p+t}) + \mu + \alpha_a + \beta_{p+t} + \gamma\_c_{+t} + \delta_{a,p+t}$. The priors for all effects are based on "second-order random walk (rw2) smoothing penalties": the prior for the age effect α_a is $f(\alpha \mid k\_\alpha) \propto k\_\alpha$^((I-2)/2) $\times \exp\{ -k\_\alpha/2 \times \Sigma$ (from i=3 to I) $[(\alpha_i - \alpha_{i-1}) - (\alpha_{i-1} - \alpha_{i-2})]^2 \}$ (with I = 17 as the number of age groups and k_α as the precision parameter); the prior for the period effect β_{p+t} is $\beta_{p+t} \mid \beta_1,...,\beta_p, k\_\beta \sim N((1+t)\beta_p - t\beta_{p-1}, k\_\beta^{-1}\times(1+2^2+\cdots+t^2))$ (with k_β as the precision parameter); and the prior for the cohort effect γ_c follows the same logic as the age effect, expressed as $f(\gamma \mid k\_\gamma) \propto k\_\gamma$^((C-2)/2) $\times \exp\{ -k\_\gamma/2 \times \Sigma$ (from j=3 to C) $[(\gamma_j - \gamma_{j-1}) - (\gamma_{j-1} - \gamma_{j-2})]^2 \}$ (with C = 34 as the number of cohort groups and k_γ as the precision parameter). All precision parameters (k_α, k_β, k_γ, k_δ) employ "weakly informative hyperpriors of Gamma (1, 0.001)", which avoid interference from subjective assumptions while ensuring reasonable smoothness of the effects. Predictive analyses were conducted using JD_GBDR (V2.22, Jingding Medical Technology Co., Ltd.) (Including R version 4.2.2, along with the following packages: BAPC (Version 0.0.36), INLA (Version 24.12.11), dplyr (Version 1.1.4), tidyr (Version 1.3.1), tibble (Version 3.2.1), purrr (Version 1.0.4), ggplot2 (Version 3.5.2), data.table (Version 1.17.0), stringr (Version 1.5.1), and magrittr (Version 2.0.3).).
